# Anti-cooperative supramolecular polymerization: a new *K*_2_–*K* model applied to the self-assembly of perylene bisimide dye proceeding *via* well-defined hydrogen-bonded dimers†
†Electronic supplementary information (ESI) available: Synthesis and characterization, NMR and UV/Vis experiments, VPO and ITC measurements, derivation of the *K*_2_–*K* model. See DOI: 10.1039/c5sc03759j



**DOI:** 10.1039/c5sc03759j

**Published:** 2015-12-02

**Authors:** Jana Gershberg, Franziska Fennel, Thomas H. Rehm, Stefan Lochbrunner, Frank Würthner

**Affiliations:** a Universität Würzburg , Institut für Organische Chemie & Center for Nanosystems Chemistry , 97074 Würzburg , Germany . Email: wuerthner@chemie.uni-wuerzburg.de; b Universität Rostock , Institut für Physik , Dynamik Molekularer Systeme , Albert-Einstein-Str. 23 , 18059 Rostock , Germany

## Abstract

A perylene bisimide dye bearing amide functionalities at the imide positions derived from amino acid l-alanine and a dialkoxy-substituted benzyl amine self-assembles into tightly bound dimers by π–π-stacking and hydrogen bonding in chloroform. In less polar or unpolar solvents like toluene and methylcyclohexane, and in their mixtures, these dimers further self-assemble into extended oligomeric aggregates in an anti-cooperative process in which even numbered aggregates are highly favoured. The stepwise transition from dimers into oligomers can not be properly described by conventional *K*_2_–*K* model, and thus a new *K*_2_–*K* aggregation model has been developed, which interpretes the present anti-cooperative supramolecular polymerization more appropriately. The newly developed *K*_2_–*K* model will be useful to describe self-assembly processes of a plethora of other π-conjugated molecules that are characterized by a favored dimer species.

## Abstract 2

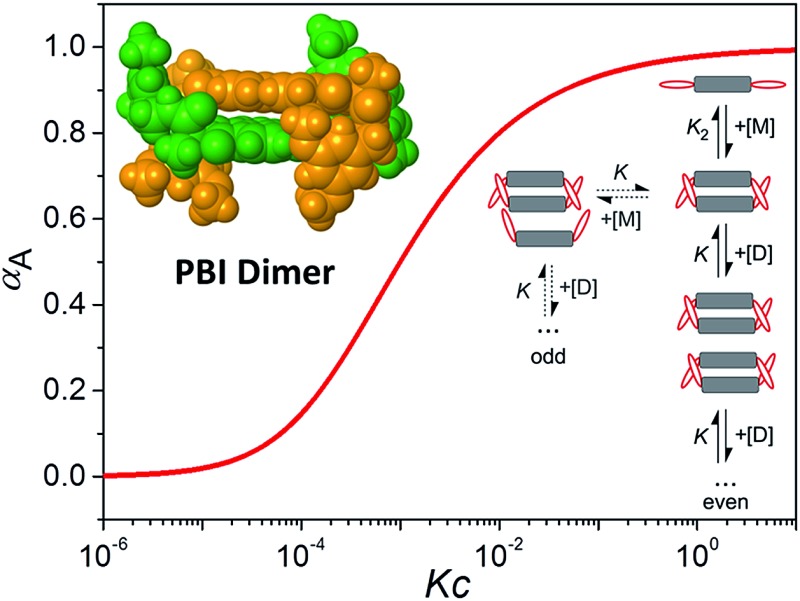
A new perylene bisimide dye self-assembles in an anti-cooperative process predominently into even numbered aggregates *via* dimers which could be interpreted by a newly developed *K*_2_–*K* model.

## Introduction

**Fig. 1:**
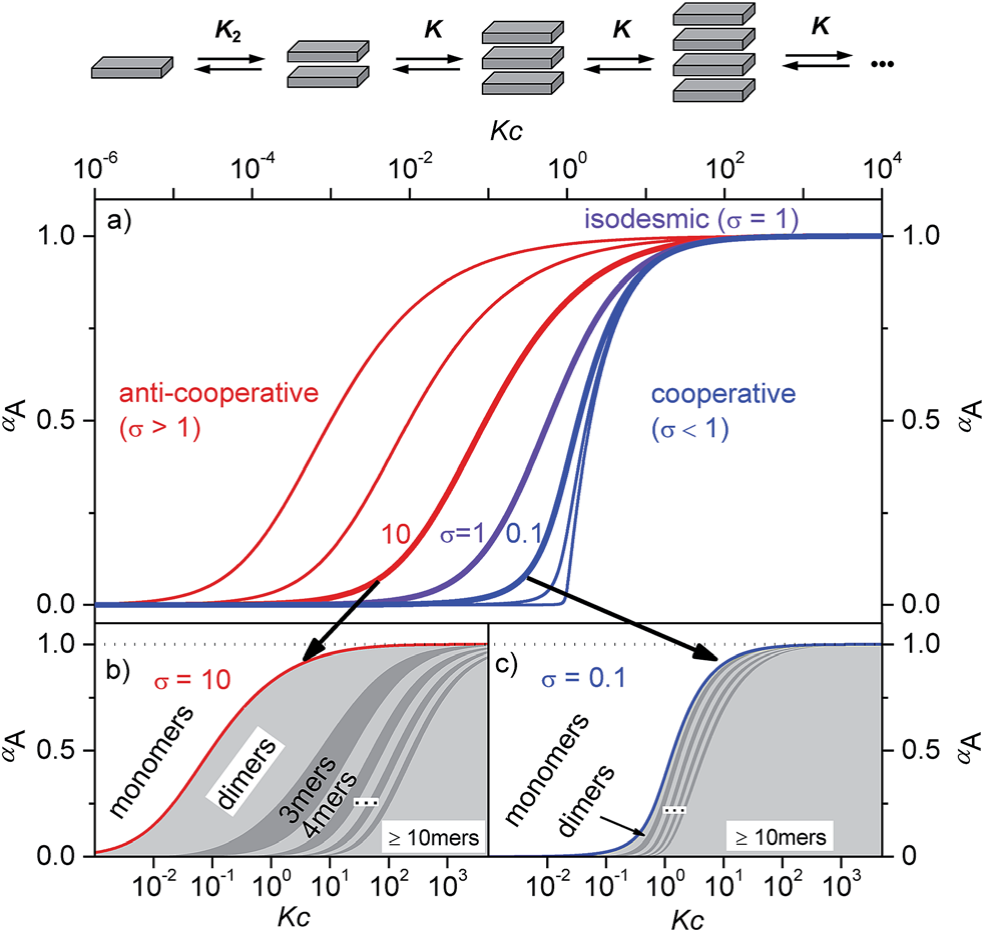
(a) Schematic illustration of a *K*_2_–*K* aggregation process. Plot of the fraction of aggregated molecules *α*_A_ as a function of the normalized concentration *Kc* with different *σ* values according to eqn (1). For the curves from left to right *σ* = 10^3^, 10^2^, 10, 1, 10^–1^, 10^–2^, and 10^–4^ were applied.^2^ (b and c) Oligomer size distribution for representative *σ* values for anti-cooperative (b, *σ* = 10) and cooperative (c, *σ* = 0.1) aggregation with a nucleus size of *s* = 2. In the anti-cooperative case a strong prevalence of the dimer is observed for a large concentration regime, whereas in the cooperative case long oligomers are formed as soon as the critical concentration *Kc* = 1 is reached.

Supramolecular polymers of (A)_*n*_-type are formed by molecules with two self-complementary binding sites when the concentration range is reached at which the interaction enthalpy between the receptor groups can overcome the entropic penalty associated with self-association.^1–3^ As π-conjugated aromatic molecules have two faces that may act as self-complementary binding sites for self-assembly by dispersion and electrostatic forces, it is thus no surprise that the majority of supramolecular polymers of such systems consist of columnar π-stacks (scheme in Fig. 1). Interestingly, for many decades the so-called isodesmic or equal *K* model (*K* = *K*_2_, violet curve in Fig. 1a and S1c†) was preferably applied to analyse concentration- and temperature-dependent supramolecular polymerizations through π–π-stacking^4^ for all kinds of dye aggregates, for instance, phthalocyanines,^5^ hexabenzocoronenes,^6^ conjugated oligomers,^7^ and perylene bisimides.^8–11^ Only about a decade ago, the generality of isodesmic growth was questioned and experimental proof was provided that numerous aggregation processes, including many of those reported previously, follow indeed a cooperative nucleation-elongation growth model in which the formation of smaller aggregates is considerably less favoured than the further elongation into extended aggregates (blue curves in Fig. 1a and c and S1a, d†).^1–3,12–16^ While the mathematical models to evaluate cooperative self-assembly processes are obviously more complicated than those applicable to isodesmic self-assembly,^17–20^ it is at least experimentally quite easy to distinguish between isodesmic and cooperative systems because the latter are characterized by a critical temperature or concentration at which monomers self-assemble within a narrow regime quite sharply into larger oligomers, see Fig. 1a and c and S1a.†

The most simple nucleation-elongation self-assembly process where aggregation affords a dimer nucleus (characterized by the dimerization constant *K*_2_), followed by more favourable further aggregation (characterized by elongation constant *K*, Fig. 1 and S1†), the so-called *K*_2_–*K* model (eqn (1)) can be applied to concentration-dependent studies.1
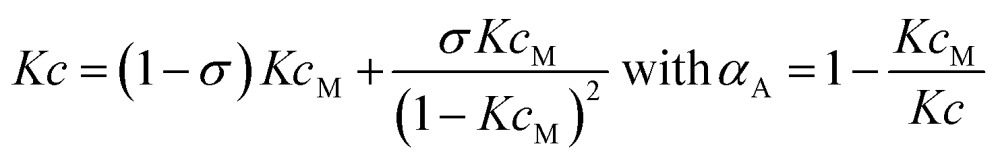
Here, *c* is the total concentration, *c*_M_ the monomer concentration, and *σ* = *K*_2_/*K* the cooperativity factor (see the section *K*_2_–*K* model in ESI† for details).^21,22^ The main physical variable which describes cooperative growth is the cooperativity factor *σ*. In order to allow a comparison between different systems the degree of aggregation is often plotted in dependence of the concentration *c* normalized with the elongation constant *K*, as this results in a degree of aggregation *α*_A_ which only depends on the cooperativity factor *σ* and is independent on the aggregation constants.

For a compound with known aggregation constants *K*_2_ and *K*, the concentration of molecules in aggregates with a size of *n*, *c*_*n*_ can then be calculated by

2 *c*_*n*_ = *nK*_2_*K*^ *n*–2^*c*_M_^*n*^

Thus, the plot of the degree of aggregation *α*_*n*_ = *c*_*n*_/*c* against the concentration leads to a continuous distribution of aggregate sizes (see in Fig. 1b, c and S1b–d†). As exemplified for *σ* = 0.1 and *σ* = 10 in Fig. 1b and c there is either an accumulation of dimers in the intermediate concentration range (Fig. 1b) or an instantaneous formation of extended aggregates (Fig. 1c).

Remarkably, while a broad variety of cooperative self-assembly processes (*σ* < 1) have been characterized in great detail in the meanwhile, work demonstrating the opposite case of anti-cooperative growth (*σ* > 1) is scarce.^14,19,23,24^ This is even more surprising because a large number of molecules are known to self-assemble into dimers for instance by hydrogen bonding,^25–27^ or electrostatically driven π-stacking of dipolar dyes.^28,29^

Accordingly, the situation of anti-cooperative growth with preferential dimerization (see area marked as dimer in Fig. 1b and S1b†) and subsequent less favourable growth into larger aggregates (denoted as grey areas) should be more prevalent than known to date. During our investigations of such an anti-cooperative aggregation process we became aware of some problems inherent in the common *K*_2_–*K* model for the case of anti-cooperative growth. For instance, for anti-cooperative aggregation with a nucleus size of two a dominance of aggregates consisting of an even number of monomers should be prevail due to the stacking of dimers. This circumstance is not covered at all by eqn (1) and (2) because these equations do not distinguish between even and odd numbered aggregates. Therefore, the development of a more elaborate model for the proper analysis of anti-cooperative *K*_2_–*K* self-assembly processes became necessary.

**Scheme 1:**
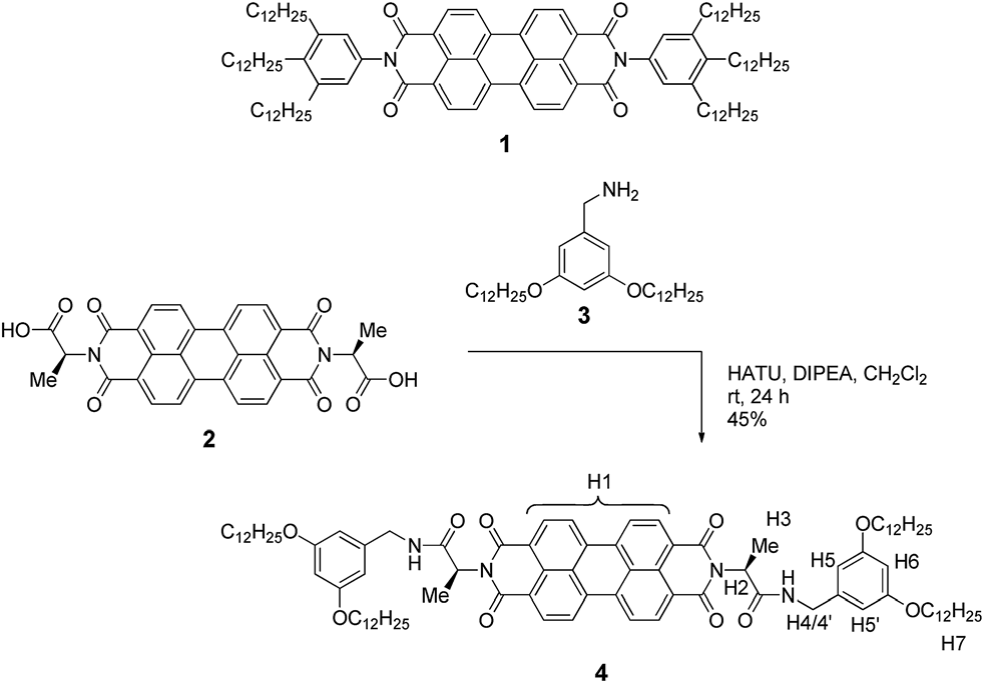
Structure of PBI **1** that showed isodesmic self-assembly,^9,11^ and synthetic route to the newly designed PBI **4** with the assignment of significant protons.

Perylene bisimide (PBI) dyes appear as good choice to derive a suitable model system for the elucidation of anti-cooperative self-assembly for many reasons. First, PBIs exhibit a pronounced aggregation strength enabling aggregate formation in dilute solutions.^30,31^ Second, their self-assembly by π–π-stacking can be easily monitored by concentration- or temperature-dependent changes in the UV/Vis absorption spectra. And third, PBI derivatives show a variable aggregation behaviour in dependence on the substituents. For instance, there are several PBI derivatives such as **1** (for structure see Scheme 1) whose aggregation has been proven to be isodesmic, *i.e.* showing equal binding constants *K* for the stepwise growth of the aggregate,^8–11^ whereas some more sophisticated structures were shown to self-assemble into dimers.^32–36^ Such PBI dimers were realized, for example, by the introduction of sterically highly demanding substituents,^32,33^ embedding of PBI dimer aggregates into the minor groove of DNA^35,36^ to prohibit further growth, and by strengthening of the dimerization by metal ion-crown ether^34^ or hydrogen-bonding interactions.^37^ Accordingly, towards our envisioned goal, and inspired by the hydrogen-bonded example of Syamakumari *et al.*,^37^ we have designed PBI **4** (Scheme 1) for which the dimerization should be privileged with regard to further aggregation by additional intermolecular hydrogen bonds between two π-stacked PBI molecules.

## Results and discussion

### Molecular design and synthesis

Our molecular design is based on the functionalization of the PBI scaffold at imide positions with hydrogen bonding amide groups derived from a homochiral amino acid and benzyl amine bearing solubilizing didodecyloxy terminal substituents. In contrast to previously investigated PBI organogelators,^15,38–41^ in which the amide groups tethered to the PBI supported a cooperative fibre growth by hydrogen bonds, in the present design a reversed connectivity of the amide group and a shortened linker unit (only one methylene unit) are applied. According to molecular modelling (see below), this design should support the formation of hydrogen-bonded dimers by intermolecular interactions between the amide NH and the PBI carbonyl groups instead of cooperative supramolecular fibre growth.

On the basis of the abovementioned concept, we have designed the homochiral PBI **4** with appended l-alanine at the imide positions and bearing dialkoxybenzyl amide substituents with dodecyl side chains, and synthesized this PBI dye according to the route displayed in Scheme 1.

The l-alanine functionalized symmetrical PBI **2** and benzyl amine **3** were prepared according to literature procedures.^32,42^ The peptide coupling reaction of amino acid functionalized PBI **2** with amine **3** in the presence of the activation reagents *N*,*N*-diisopropylethylamine (DIPEA) and *O*-(7-azabenzotriazol-1-yl)-*N*,*N*,*N*′,*N*′-tetramethyluronium hexafluorophosphate (HATU) afforded the desired PBI building block **4** in 45% yield. This new compound was properly characterized by ^1^H NMR, high-resolution mass spectroscopy and elemental analysis. For the synthesis procedure and product characterization data see the ESI.†

### Self-assembly of PBI **4** into dimers in chloroform

NMR spectroscopy is a powerful technique to follow the formation of supramolecular structures qualitatively and quantitatively as well. Thus, we have first studied the self-assembly of PBI **4** by concentration-dependent ^1^H NMR spectroscopy in chloroform. Upon increasing the concentration of **4** from 6.5 × 10^–6^ M to 1.3 × 10^–2^ M, the signal (a broad singlet) of the perylene protons (H1) experiences a significant upfield shift from 8.7 to 7.7 ppm, indicating π–π-interactions between the PBI dyes (Fig. S2†).^32,43,44^ In contrast, the signal of the amide NH proton (partially overlapped by the solvent signal) undergoes a downfield shift upon increasing the concentration, pointing at the formation of hydrogen bonds.^45–47^

**Fig. 2:**
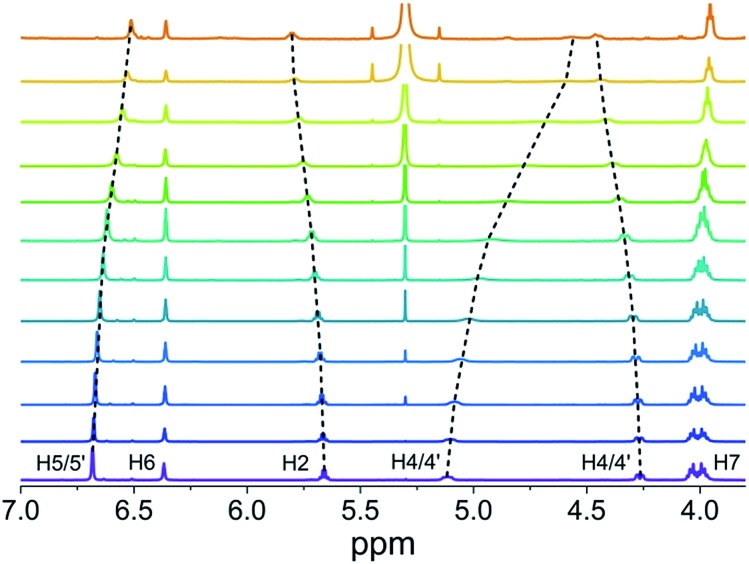
Changes of the chemical shifts of PBI **4** protons in concentration-dependent ^1^H NMR spectra in CDCl_3_ at 298 K (1.3 × 10^–2^ M (bottom) to 6.5 × 10^–6^ M (top)).

The chemical shifts of the remaining more peripheral protons experience comparatively smaller changes (Δ*δ* < 0.2 ppm) upon concentration change (Fig. 2). For instance, protons H5/5′ and H7 in the terminal group show a small downfield shift at higher concentration which is suggestive of very weak C–H···O hydrogen bonding to the carbonyl oxygen atom, while the small upfield shift of H2 and H6 protons may relate to an aromatic shielding effect by the neighbouring PBI dye upon π–π-stacking. At higher concentration (1.3 × 10^–2^ M), the hydrogen bonding of the amide groups provoked a splitting of the diastereotopic protons H4 and H4′ into two well separated signals (Fig. 2). The sharp signals observed in ^1^H NMR spectra of PBI **4** over the measured concentration range suggest the formation of a π-stack of discrete size. Nonlinear least-square analysis of the changes of the proton resonance signals shows a good fit to the monomer–dimer model with a dimerization constant *K*_2_ in the range of about 1.2 × 10^4^ M^–1^ (Fig. S3†). This high dimerization constant suggests an almost complete dimer formation of PBI **4** in chloroform at the highest utilized concentration of 1.3 × 10^–2^ M, *i.e.* a degree of aggregation *α*_A_ of 93% is calculated (see Table S1†). Compared with the binding constants of other known PBIs in chloroform, *e.g.* <20 M^–1^ for PBI **1**,^11^ the dimerization constant for PBI **4** is indeed very high, which we attribute to a favourable interplay of π–π-interactions and hydrogen bonds.^11^ We note that the involvement of hydrogen bonds in the dimer aggregates formed in chloroform was confirmed by IR spectroscopy (Fig. S4,† left panel).^37^

**Fig. 3:**
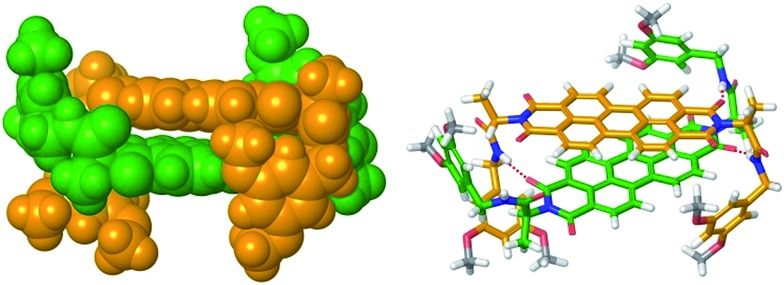
OPLS2001* (MacroModel) geometry-optimized structure of self-assembled PBI **4** dimer with dodecyloxy chains replaced by methoxy groups.

Furthermore, the size of the PBI **4** aggregate formed in chloroform at the concentration of 1.3 × 10^–2^ M could be estimated from diffusion ordered (DOSY) NMR spectrum (Fig. S5,† left panel). From the diffusion coefficients, a hydrodynamic diameter of 2.6 nm was determined for the PBI **4** aggregate according to the Stokes–Einstein equation (see eqn (S7) in ESI†). By measuring the distance between the most distant atoms in the OPLS2001* (MacroModel) geometry optimized dimer structure (bearing methoxy instead of dodecyloxy substituents), an averaged size of the PBI **4** dimer of 1.8 nm was identified (Fig. 3). Taking into account the length of the dodecyl chains of ∼1 nm, this size is in good agreement with that estimated by DOSY NMR experiments. We note that our vapour pressure osmometry (VPO) measurements in chloroform (Fig. S6†) likewise corroborate the presence of dimer aggregates of PBI **4** in chloroform in the applied concentration range (for details see ESI†).

To further substantiate the formation of dimers, we performed rotating-frame nuclear Overhauser effect (ROESY) NMR experiments with a sample of PBI **4** in chloroform at the highest concentration (1.3 × 10^–2^ M) applied for the ^1^H NMR studies. If dimers were formed, cross-coupling between the protons of two PBI molecules in close vicinity should be observed. Indeed, three signals are observed in the ROESY NMR spectrum (Fig. S7†) for the intermolecular cross-coupling of protons H2 and H5/5′, H2 and NH, and H3 and H5/5′ (marked with red circles in the spectrum). These results also corroborate the formation of dimers.

As mentioned before, geometry-optimized dimer structure of PBI **4** was modelled by OPLS2001*. The analysis of the dimer structure (Fig. 3) shows that the two PBI molecules with an interplanar distance of 3.3 Å, twist angle of 40° and slip angle of 83° form a bracket-like structure, in which the phenyl residues of the individual molecules clasp the counter molecule, forming hydrogen bonds between NH protons and carbonyl oxygen atoms of the other PBI molecule. According to the modelled dimer structure, the distances between the cross-coupled protons are close to the 5 Å limit for ROESY experiments. The rigid dimer structure obtained from the molecular modeling illustrates well that the phenyl rings and their long alkoxy chains shield the PBI cores, thus further π–π-stacking into larger aggregates becomes unfavored.

**Fig. 4:**
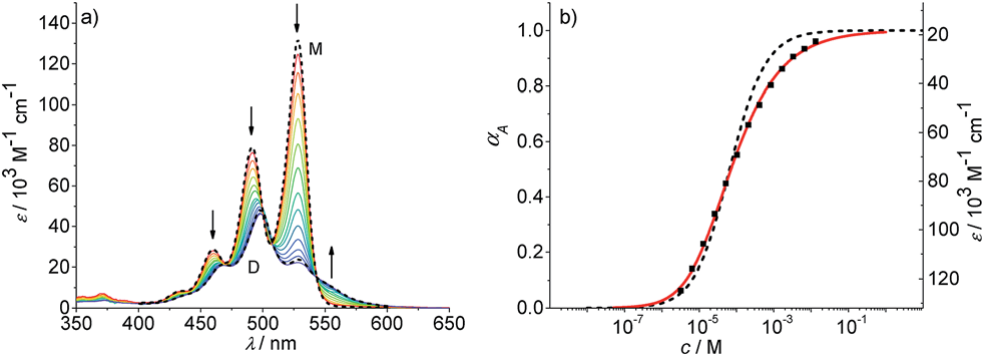
(a) Concentration-dependent UV/Vis absorption spectra of PBI **4** in chloroform (*c* = 3.2 × 10^–6^ to 1.3 × 10^–2^ M) at 293 K. The dotted lines are the calculated monomer (M) and dimer (D) spectra from available data in terms of the dimer model. Arrows indicate the spectral changes upon increasing concentration. (b) Analysis of the concentration-dependent extinction data at 528 nm according to the isodesmic (dashed black line) and dimer (red line) aggregation models (*R* ^2^ = 0.999). Left and right axes display the degree of aggregation and extinction, respectively, for a direct comparison.

We have further studied the self-assembly of PBI **4** by the commonly used concentration-dependent UV/Vis spectroscopy. Due to the good solubility of PBI **4** in chloroform, absorption studies could be performed over a wide concentration range from 3.2 × 10^–6^ to 1.3 × 10^–2^ M. At low concentrations, the spectra show a well-resolved vibronic structure with the 0–0, 0–1 and 0–2 transitions of the monomeric PBI molecule with the three most intensive bands at 528, 491 and 460 nm (Fig. 4).

The ratio of *A* ^0→0^/*A* ^0→1^ of the vibronic transitions of the lowest energy absorption band at the lowest concentration (3.2 × 10^–6^ M) is larger than 1.6, which is a characteristic spectral feature of monomeric bay-unsubstituted PBI dyes in solution.^48,49^ Upon increasing the concentration, a transformation to a broadened spectrum with a significantly lower extinction coefficient at 498 nm occurs. The hypsochromic shift of the absorption maximum of 30 nm indicates a predominant H-type excitonic coupling, which was observed for many other bay-unsubstituted PBI dyes.^8,9,33^ Over the whole concentration range two clear isosbestic points at 543 and 507 nm are evident, indicating an aggregation equilibrium between two species. Nonlinear least-squares analysis of the concentration-dependent extinction coefficients at 529 nm using the monomer–dimer model (eqn (3)) reveals a good fit (Fig. 4b and ESI†).3
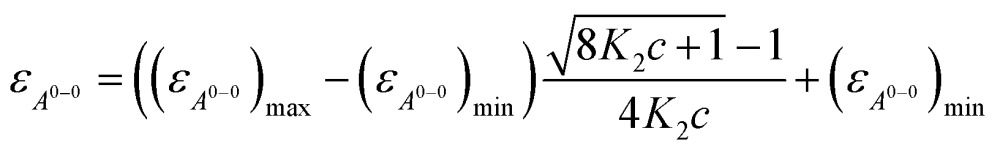
where (*ε*_*A*^0–0^_)_max_ and (*ε*_*A*^0–0^_)_min_ are the maximum and minimum extinction coefficients as calculated by this analysis for the extinction of the monomer and dimer respectively, and *ε*_*A*^0–0^_ is the respective concentration-dependent extinction.

With the obtained values for (*ε*_*A*^0–0^_)_max_ and (*ε*_*A*^0–0^_)_min_ the degree of aggregation *α*_A_ was calculated according to eqn (4),4
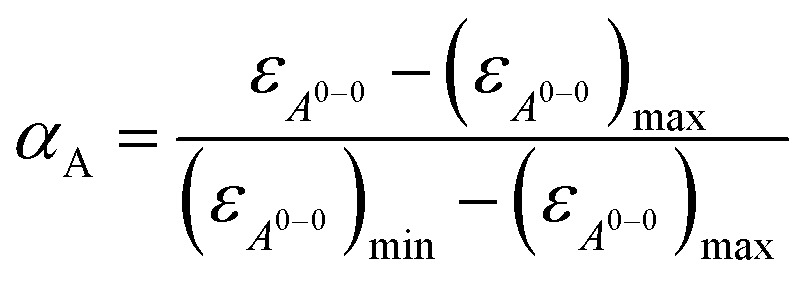



We like to note that the calculation of *α*_A_ as denoted in eqn (4) is only possible when only two distinct species exist. These species have to be distinguishable by clear different extinction values at a given wavelength.

By the nonlinear least-squares analysis of the experimental extinction coefficients a dimerization constant *K*_2_ of 1.41 × 10^4^ M^–1^ ± 3.2 × 10^2^ M^–1^ and a degree of dimerization *α*_A_ from 6% to 96% were calculated in the considered concentration regime between 1.6 × 10^–6^ and 1.3 × 10^–2^ M, indicating an almost complete transition from monomers to the dimer aggregate of PBI **4**. These results are in excellent agreement with those of ^1^H NMR studies discussed before. Additionally, the calculated absorption spectra of monomer and dimer match very well with the respective experimental spectra (Fig. 4a, black dashed lines).

To obtain some thermodynamic parameters for the dimerization process of PBI **4**, isothermal calorimetry (ITC) dilution experiments were performed in chloroform as well (Fig. S8†). These experiments revealed an enthalpy (Δ*H*°) value of 36.5 kJ mol^–1^ ± 1.5 kJ mol^–1^ for the dimer dissociation process. Accordingly, the dimerization of PBI **4** should be an exothermic process as expected for hydrogen-bonding and π–π-stacking aggregation, which are both enthalpically favored processes. It is to note that the dimerization constant (≈10^4^ M^–1^) obtained from ITC experiments agrees again well with the values obtained from NMR and UV/Vis experiments.

### Self-assembly in toluene, methylcyclohexane and mixtures of methylcyclohexane and toluene

In the previous concentration-dependent UV/Vis spectroscopic studies in chloroform only dimers of PBI **4** could be observed within the explored concentration range. This can be rationalized by the fact that all available amide NH units are involved in the formation of the dimer, hence further aggregate growth is not supported anymore by hydrogen bonding, and/or even hindered by the orientation of the didodecyloxyphenyl substituents in the bimolecular complex (see Fig. 3). Our previous work on the aggregation of PBI dyes by only π–π-stacking interactions suggested that the binding strength can be considerably increased by reducing the solvent polarity. For instance, aggregation constants of *K* < 20 M^–1^ in chloroform, *K* = 590 M^–1^ in toluene, and *K* = 9.7 × 10^4^ M^–1^ in methylcyclohexane were determined for the non-hydrogen bonding PBI **1**.^11^

Therefore, we performed aggregation studies of PBI **4** in less polar toluene by concentration-dependent (*c* = 2.0 × 10^–7^ to 1.3 × 10^–2^ M) UV/Vis spectroscopy and observed similar H-type aggregates as in chloroform (Fig. S9a†). However, the spectra in toluene do not show isosbestic points over the whole concentration range, indicating that longer aggregates than the dimer are present. The existence of longer aggregates is also supported by VPO and DOSY NMR experiments (see Fig. S5 and S10† for details). However, the hydrogen bonded aggregates (see FT-IR in Fig. S4,† right panel) remain of small size. A good fit of *α*_A_ values obtained from the monomer absorption maximum at 529 nm is obtained with the common *K*_2_–*K* model^21,22^ with the nucleus size of *s* = 2, a *σ* value of 50 and a relatively small aggregation constant of *K* = 2.20 × 10^3^ M^–1^ (Fig. S9b†). From *σ* = *K*_2_/*K*, a dimerization constant *K*_2_ of 1.10 × 10^5^ M^–1^ is calculated, meaning that dimers are more easily formed than larger aggregates. In order to enhance the aggregation further a less polar solvent is required. As the dimer is highly favored for PBI **4**, it is expected that even numbered aggregates prevail. Unfortunately, such a process cannot properly be described by the conventional *K*_2_–*K* model (eqn (1) and (2)), because this model does not consider the differentiation between the even and odd numbered aggregates. Therefore, the development of a new model that considers such differentiation is in demand in order to properly evaluate such self-assembly processes.

**Fig. 5:**
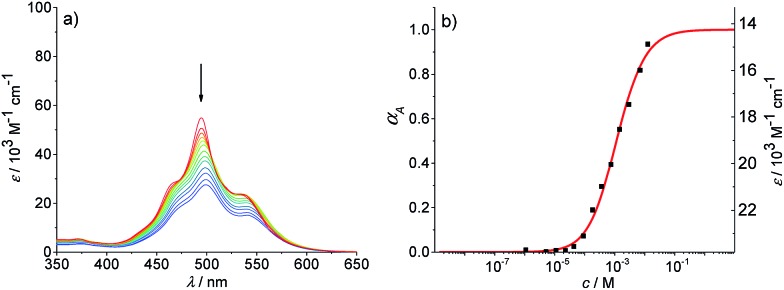
(a) Concentration-dependent UV/Vis absorption spectra of PBI **4** in methylcyclohexane (*c* = 1.1 × 10^–6^ to 1.3 × 10^–2^ M) at 293 K. Arrows indicate the spectral changes upon increasing concentration. (b) Analysis of the absorption data according to isodesmic model at 530 nm (*R* ^2^ = 0.995).

To promote the growth of larger aggregates, we performed aggregation studies in nonpolar methylcyclohexane and mixtures of methylcyclohexane and toluene. Absorption studies were first conducted in methylcyclohexane in the concentration range of 1.1 × 10^–6^ to 1.3 × 10^–2^ M (Fig. 5a). At the highest concentration (1.3 × 10^–2^ M^–1^), a broad spectrum with a maximum at 499 nm is observed. Upon decreasing the concentration, the spectral features remain unchanged but experience a slight hypsochromic shift to 495 nm and a hyperchromic effect (increase of absorbance for smaller aggregates). Even at low concentrations, no transition to the spectrum of PBI **4** monomers is observed. This indicates that a large amount of hydrogen-bonded dimers (structure is probably similar to that shown in Fig. 3) seems to exist even at the lowest concentration. These dimers then grow into larger (even numbered) aggregates upon increasing concentration.^50^ One option to characterize this aggregation behavior is to assume a complete dimerization and to describe the further aggregation by an isodesmic growth with the dimer as the smallest repeat unit. Based on these assumptions, the absorption data at 530 nm in methylcyclohexane could be fitted according to the isodesmic model (Fig. 5b). The degree of aggregation increases with increasing concentration from 1% to 93%, and a binding constant *K* of 1030 M^–1^ ± 50 M^–1^ is obtained for the π–π-stacking of the PBI H-dimers into larger aggregates. It is noteworthy that such analysis anticipates that all larger oligomers are composed of dimeric units, hence the aggregates are of even numbered size. However, the analysis of the absorption data at some other wavelengths revealed a plateau for the extinction coefficients in the concentration range from 1 × 10^–5^ to 5 × 10^–5^ M (for representative example see Fig. S11a†). Such a plateau is indicative for more complex aggregation behaviour and shows that the assumption of a complete dimerization at low concentration is wrong and therefore the description with the isodesmic model fails (see Fig. S11a†). Consequently, we tried to analyse the data with a successive description by the dimer model for low concentrations (Fig. S11b†) and the isodesmic model for higher concentrations, where the dimer is considered to be the smallest repeat unit (Fig. S11c†). This fitting procedures lead to a dimerization constant *K*_2_ of 8.3 × 10^5^ M^–1^ ± 7 × 10^4^ M^–1^ and to an elongation constant *K* of 890 M^–1^ ± 50 M^–1^. This kind of successive analysis is obviously prone to significant errors, because the concentration regimes for dimerization and elongation are not sufficiently separated. Especially, for the sub-step of dimerization the analysis is crude since only a small part of the dimerization process (range from 23–83%) is covered. Desirable for a proper analysis is an environment where the whole range from monomers to long aggregates is observable.

**Fig. 6:**
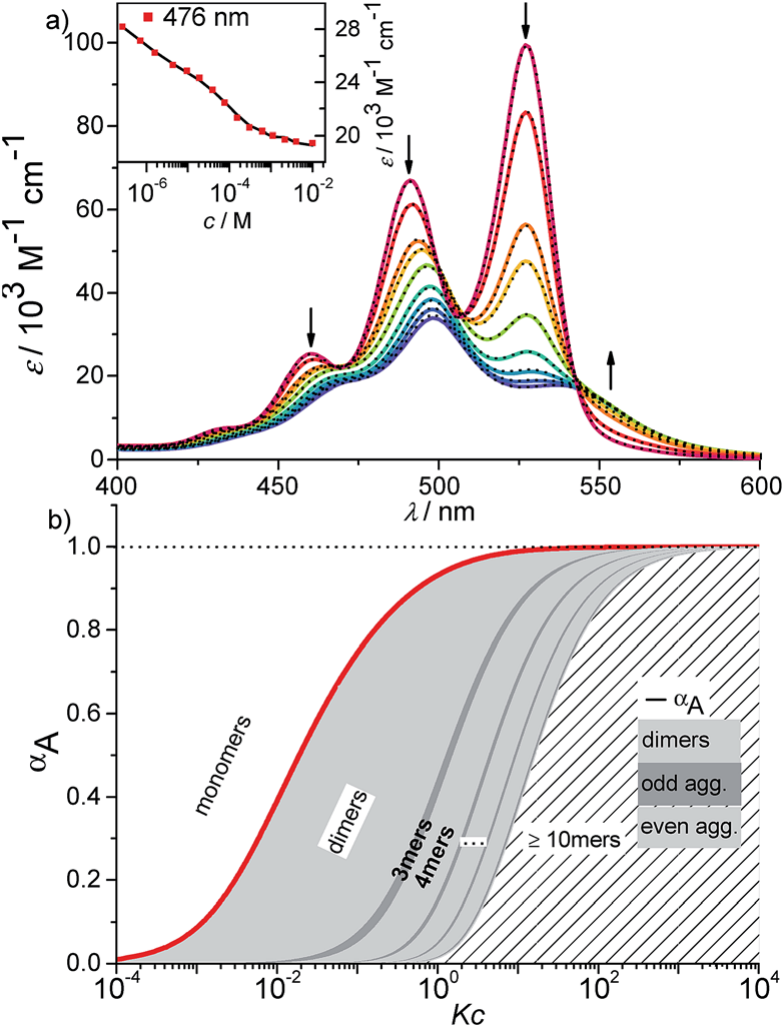
(a) Concentration-dependent UV/Vis absorption spectra (coloured lines) and the spectra reconstructed by the new *K*_2_–*K* model for anti-cooperative supramolecular polymerization (black dashed lines) of PBI **4** in a methylcyclohexane/toluene (30 : 70) mixture (*c* = 2.7 × 10^–7^ to 1.0 × 10^–2^ M) at 293 K, the complete data set is shown in the ESI.† Arrows indicate the spectral changes upon increasing concentration. Inset: comparison between fit (black line) and the concentration dependent extinction at the wavelength of 476 nm (red symbols) for stepwise aggregation of PBI **4**. (b) Degree of aggregation *α*_A_ (red line) and the size distribution of *n*-mer aggregates of PBI **4** (light grey for even numbered aggregates and dark grey for odd numbered aggregates) calculated according to the new anti-cooperative *K*_2_–*K* model (eqn (8) and (9)). Even numbered aggregates dominate in the sample as expected.

As an ideal solvent to cover the entire range of species from monomers to extended aggregates, a particular mixture of toluene and methylcyclohexane should be most appropriate. Therefore, the absorption spectra of PBI **4** in various mixtures of methylcyclohexane and toluene at a concentration of 1.6 × 10^–6^ M were analyzed, which displayed the expected transition from the monomeric PBI in pure toluene to the aggregated species for 90% methylcyclohexane content (Fig. S13†). A 30 : 70 mixture of methylcyclohexane/toluene proved to be the best suitable solvent for further investigations. At the lowest concentration of 2.7 × 10^–7^ M in this solvent mixture, the spectrum displayed the characteristic vibronic structure of the monomeric species (*A* ^0→0^/*A* ^0→1^ = 1.5 for the two lowest energy vibronic transitions) with three prominent bands at 528, 491 and 460 nm (Fig. 6a and S15,† top panel). With increasing concentration up to 1.3 × 10^–2^ M, the spectrum of the aggregated species appears with the hypsochromically shifted maximum at 498 nm. The analysis of the absorption data at distinct wavelengths again showed a plateau region at intermediate concentrations similar to the observations made in pure methylcyclohexane (for representative example see inset in Fig. 6a). By the separate fitting of the data for the two regimes, *i.e.* by the monomer–dimer model for the formation of dimers and the isodesmic model for the aggregation of the dimers to larger assemblies, a value for *K*_2_ of 1.13 × 10^6^ M^–1^ ± 2.3 × 10^4^ M^–1^ and for *K* of 1.80 × 10^4^ M^–1^ ± 600 M^–1^ were determined (Fig. S14†).

### *K*_2_–*K* model for anti-cooperative supramolecular polymerization

Clearly, the so far applied method for the data analysis is of unsatisfactory accuracy and is completely useless if the two processes are not sufficiently separated as it is the case for weak anti-cooperativity which is probably the most common situation for the majority of dye aggregation processes. Likewise, as already pointed out, the conventional *K*_2_–*K* model is also not suitable because it does not distinguish between more favoured even and less favoured odd aggregate species. Thus, to evaluate anti-cooperative supramolecular polymerization processes *via* the most common dimer intermediates and subsequent growth into even numbered aggregates, an appropriate mathematical model had to be derived. For this purpose, we elaborated a model which is based on the *K*_2_–*K* model but distinguishes between even and odd numbered aggregates (for details see ESI†).

Dimers are formed by the assembly of two monomers M with a dimerization constant *K*_2_:

5 M + M ⇌ D;  [M_2_] = *K*_2_[M]^2^

where [M] = *c*_M_ is the monomer and [M_2_] = *c*_D_ the dimer concentration. The π-surfaces of the dimers are shielded by the substituents, and hence leading to a less favorable aggregation with an elongation constant *K*. The dimer can be extended either with another monomer, leading to trimers with a trimer concentration [M_3_] of:

6 D + M ⇌ M_3_;  [M_3_] = *K*[M_2_][M]

or with another dimer, leading to tetramers with a concentration [M_4_] of:

7 D + D ⇌ M_4_;  [M_4_] = *K*[M_2_]^2^

Further association leads to8
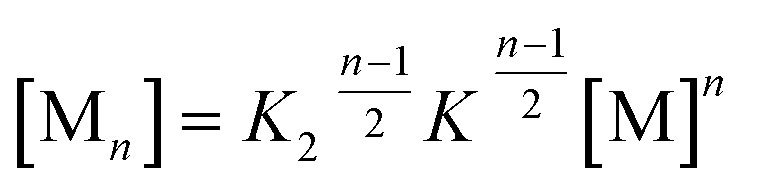
for odd numbered aggregates and to9
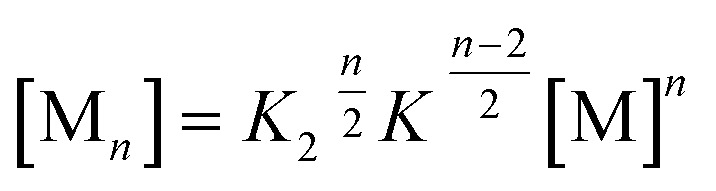
for even numbered aggregates. These equations indicate that as soon as an aggregation degree is reached where dimers dominate with respect to monomers the formation of even numbered aggregates is favoured due to *K*_2_ > *K*.

If the sum over all odd, as well as for all even *n* is performed, the resulting concentration of molecules in odd aggregates is given by10
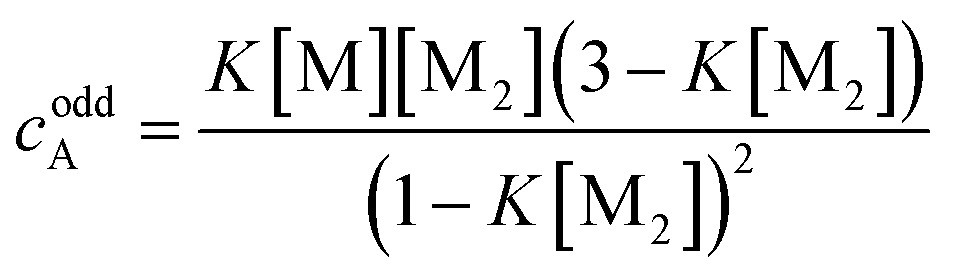
and for molecules in even aggregates (including dimers) by11
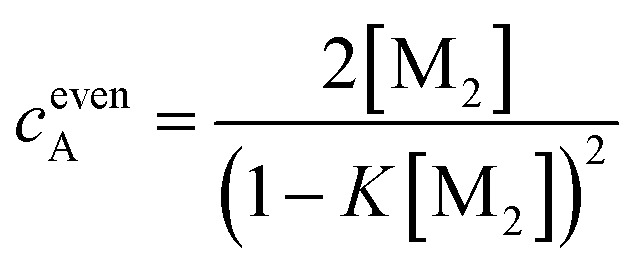



The total concentration of molecules is then given by

12 *c* = *c*_M_ + *c*oddA + *c*evenA

This model is fitted globally to the absorption data of PBI **4** in the solvent mixture methylcyclohexane/toluene 30 : 70 and thereby the aggregation constants *K*_2_ and *K* as well as the absorption spectra of monomer, dimer and oligomer are extracted. Details on the fitting procedure are given in the ESI.†

By using this modified *K*_2_–*K* model for anti-cooperative supramolecular polymerization, the whole data set at 476 nm (see inset in Fig. 6a) as well as for all other wavelengths (Fig. S15,† bottom panel) can be fitted perfectly, covering the complete curves formed by the experimental data points. Additionally, the measured extinction at each concentration is in very good agreement with the extinction calculated by the newly derived model (Fig. 6a, black dashed lines; for complete data set see Fig. S15,† top panel). It has to be noted that the calculation of *α*_A_ from the spectra by means of eqn (4) is not possible in this case due to the fact that three different species (monomers, dimers, larger aggregates) with significant different extinction contribute to the spectra.

**Fig. 7:**
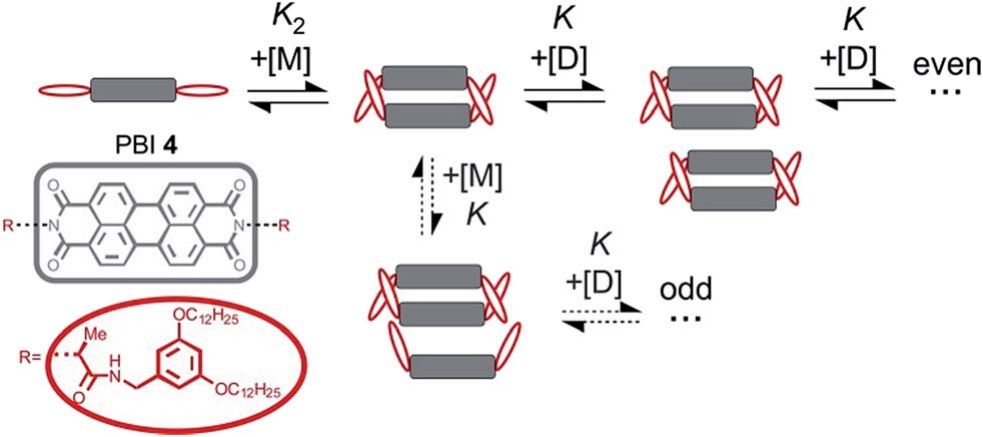
Schematic illustration of the new model for supramolecular polymerization which describes the aggregation pathway of PBI **4**.

The calculated dimerization constant *K*_2_ of 4.5 × 10^5^ M^–1^ and the elongation constant *K* of 1.1 × 10^4^ M^–1^ are in the same range as the ones obtained before by separating of the 476 nm data into two ranges, but now the aggregation process is described in total. Furthermore, our new model provides insight into the ratio of odd and even numbered aggregates, *e.g.* the calculated amount of odd numbered aggregates at the highest concentration of 1.0 × 10^–2^ M is about 16% (eqn (S28)†) and even numbered aggregates accordingly indeed prevail (∼84%) as expected. From the plot of *α*_*n*_ as a function of *Kc* the distribution of the distinct species in methylcyclohexane/toluene mixture can be obtained (Fig. 6b and S16†). Starting from the lowest concentration, the amount of monomers decreases to zero while the amount of the favoured dimer species accumulates at intermediate concentrations up to >60%. At higher concentrations, the growth into larger aggregates starts, leading to a columnar π-stack. In such stack each dye molecule has a more close (supported by hydrogen bonding and π–π-stacking) and a distant neighbour (just π–π-stacking). Fig. 7 illustrates the proposed aggregation pathway and the prevalence of even numbered aggregates. Due to the accumulation of dimers, the formed oligomers consist mainly of an even number of molecules resulting in an alternating distribution of even and odd numbered aggregates (see light and dark grey areas in Fig. 6b and the histogram in Fig. S16†). The newly developed *K*_2_–*K* model for anti-cooperative supramolecular polymerization is capable to describe the prevalence of even numbered aggregates which is obviously not possible by the conventional *K*_2_–*K* model which exhibits a continuous size distribution for anti-cooperative aggregation (see Fig. 1b and S1b†).

**Fig. 8:**
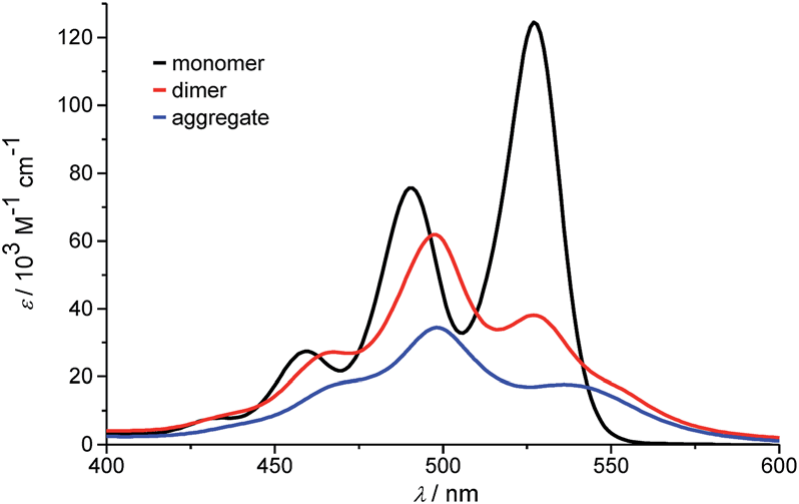
Calculated monomer, dimer and aggregate spectra of PBI **4** according to the new anti-cooperative *K*_2_–*K* model.

At the highest concentration of our study (1.0 × 10^–2^ M), a broad distribution of dominantly even numbered oligomers exists and only <20% of the molecules are found in small stacks composed of less than 10 units (see shaded area in Fig. 6b). Additionally, the global analysis of the experimental spectra with the anti-cooperative aggregation model gives the distinct spectra of monomer, dimer and extended aggregates (Fig. 8).

The spectrum of the monomer shows the characteristic vibronic structure, while the ones of the dimer and longer aggregates display significant differences. The dimer spectrum still exhibits significant vibronic structure, which seems to vanish in the aggregate spectrum. This is an important finding and may be rationalized by less defined contacts between the respective dimers, *i.e.* a broader distribution of translational and rotational displacements for the non-hydrogen-bonded contacts (*i.e.* disorder) compared to the structurally more homogeneous hydrogen-bonded dimers. Moreover, the extinction coefficient of dimers per chromophore is obviously higher than the one of the larger aggregate species, which is in accordance with the theory of hypochromism that was derived to explain this effect for π-stacked oligonucleotides in DNA already half a century ago.^51,52^ With the new *K*_2_–*K* model for anti-cooperative supramolecular polymerization, we can now also analyze the concentration-dependent absorption spectra obtained in pure toluene. Even though the analysis of the data with the simple *K*_2_–*K* model (as used for cooperative supramolecular polymerization) provided a quite good fit, the resulting distribution with equal amounts of odd and even numbered species left us unsatisfied (see above). In this regard, the outcome of our analysis employing the new *K*_2_–*K* model is quite pleasing because the obtained dimerization constant *K*_2_ of 9.8 × 10^4^ M^–1^ and elongation constant *K* of 1.9 × 10^3^ M^–1^ are indeed quite similar to those determined by the conventional *K*_2_–*K* model. Again the amount of odd numbered aggregates is below 14% over the whole investigated concentration range in toluene according to our advanced analysis. The calculated distribution of species in toluene shows that small aggregates up to hexamers prevail in the investigated concentration range up to 1.3 × 10^–2^ M (data not shown).

## Conclusions

In this paper, an anti-cooperative supramolecular polymerization process was elucidated for the first time in detail from both the experimental and theoretical point of view. For this purpose, a new perylene bisimide dye (PBI **4**) was designed and synthesized, which shows a strong preference for the formation of dimers by the assistance of hydrogen bonds from the alanine derived amide function at the imide position to the carbonyl groups.

For this PBI dye, the aggregation process from monomers *via* dimers to larger oligomers was unambiguously characterized by a broad range of spectroscopic techniques which also disclosed the responsible driving forces for the formation of these aggregates, *i.e.* π–π-stacking interactions and hydrogen bonds. For the correct description of the stepwise aggregation process with increasing concentration *via* dimers, tetramers, hexamers, *etc.* a new *K*_2_–*K* model for anti-cooperative supramolecular polymerization was developed which afforded a comprehensive description of the anti-cooperative stepwise association pathway of this PBI dye with unprecedented insights into the species distribution and the spectral differences of dimers and larger aggregates. Whilst our PBI molecule was especially tailored to provide a convincing showcase for an anti-cooperative supramolecular polymerization we like to advertise our new model also for any other anti-cooperative supramolecular polymerization process that pursues *via* a favored dimer species.
