# Supplementary material for: Anti-cooperative supramolecular polymerization: a new K 2–K model applied to the self-assembly of perylene bisimide dye proceeding via well-defined hydrogen-bonded dimers
Source: Chem Sci. 2015 Dec 2;7(3):1729–37. doi: 10.1039/c5sc03759j (PMC5582317; doi:10.1039/c5sc03759j)
Supplement: Supplementary file 1 [file SC-007-C5SC03759J-s001.pdf]

Electronic Supplementary Material (ESI) for Chemical Science.
This journal is © The Royal Society of Chemistry 2015

**Electronic Supplementary Information for**

# Anti-cooperative supramolecular polymerization: a new $K_2$-$K$ model applied to the self-assembly of perylene bisimide dye proceeding via well-defined hydrogen-bonded dimers

Jana Gershberg,[a] Franziska Fennel,[b] Thomas H. Rehm,[a] Stefan Lochbrunner,[b] Frank Würthner[a]*

[a]*Universität Würzburg, Institut für Organische Chemie & Center for Nanosystems Chemistry, Am Hubland, 97074 Würzburg, Germany. E-mail: wuerthner@chemie.uni-wuerzburg.de; Fax: +49 931 3184756; Tel: +49 931 3185340.* [b] *Universität Rostock, Institut für Physik, Dynamik Molekularer Systeme, Albert-Einstein-Str. 23, 18059 Rostock, Germany.*

**Table of Contents**

1. Materials and Methods S-2
2. Schematic illustration of a $K_2$-$K$ aggregation process S-3
3. Synthesis of PBI **4** S-4
4. Concentration-dependent $^1$H NMR spectra of PBI **4** in chloroform S-4
5. Application of the monomer-dimer model on $^1$H NMR data S-5
6. FT-IR measurements in chloroform and toluene S-6
7. DOSY NMR experiments in chloroform and toluene S-6
8. VPO measurements in chloroform S-7
9. ROESY NMR experiments in chloroform S-8
10. Isothermal titration calorimetry experiments in chloroform S-9
11. Concentration-dependent UV/Vis spectra of PBI **4** in toluene S-9
12. Conventional $K_2$-$K$ model S-10
13. VPO measurements in toluene S-10
14. Analysis of absorption data of PBI **4** in methylcyclohexane at 502 nm S-11
15. DOSY NMR experiments in methylcyclohexane S-12
16. Absorption spectra of PBI **4** in methylcyclohexane/toluene mixtures S-12
17. Analysis of absorption data of PBI **4** in a methylcyclohexane/toluene (30:70) mixture at 476 nm S-13
18. $K_2$-$K$-model for anti-cooperative supramolecular polymerization S-13
19. Fit algorithm to analyze the anti-cooperative supramolecular polymerization S-15
20. Analysis of the concentration-dependent UV/Vis spectra of PBI **4** in a methylcyclohexane/toluene mixture of 30:70 by the $K_2$-$K$ model for anti-cooperative supramolecular polymerization S-17
21. HRMS-ESI and $^1$H NMR spectra of PBI **4** S-19
22. References S-21

## 1. Materials and Methods

Solvent and reagents were purchased from commercial sources, unless otherwise stated, and purified and dried according to standard procedures.[1] Reactions were monitored by TLC on silica gel plates (Merck TLC silica gel 60 F254 aluminum sheets). All perylene bisimide derivatives are colored compounds, thus no additional visualization of the spots was necessary. Column chromatography was performed on silica gel (MerckSilica 60, particle size 0.04 – 0.063 mm). Gel permeation chromatography was performed on a Shimadzu Recycling GPC system (LC-20AD prominence pump; SPDMA20A, prominence diode array detector) using three preparative columns (JAIGEL-1H, JAIGEL-2H und JAIGEL-2.5H) from Japan Analytical Industries Co., Ltd. and chloroform as eluent. NMR experiments were conducted on a Bruker Avance 400 or Bruker DMX 600 spectrometer with TMS or residual undeuterated solvent as internal standard. The chemical shifts are reported in ppm relative to TMS or residual undeuterated solvent as internal standard ($\delta$ scale). The apparent coupling constants *J* are given in Hertz (Hz). The following abbreviations are used to describe the signal fine structure: s = singlet, d = doublet, t = triplet, q = quartet, quint = quintet, dd = doublet of doublets, m = multiplet, and bm = broad multiplet. Melting point was measured on a polarization microscope BX41 of Olympus equipped with MGW Lauda RM6 cooling systems and is uncorrected. All FT-IR spectra were measured on a FT/IR-4100 spectrophotometer (Jasco). High-resolution electrospray ionization (ESI) mass spectra were measured on a MicroTOF Focus instrument (Bruker Daltronik GmbH).

The UV/Vis absorption spectra were recorded in conventional quartz cells of appropriate path length on a Perkin Elmer Lambda 35 spectrophotometer equipped with a Peltier system for temperature control. Chloroform and toluene of spectroscopic grade were used for the measurements.

The vapor pressure osmometry (VPO) measurements were performed at 303 K on a KNAUER osmometer with a universal temperature measurement unit. Benzil was used as standard to generate calibration curves in terms of *R* (ohm) vs. molal osmotic concentration in the respective solvent.

Isothermal titration calorimetry (ITC) measurements were recorded on a VP-ITC instrument from MicroCal. Dilution ITC experiments were performed by sequential injections of a concentrated solution of PBI **4** ($7.5 \times 10^{-3}$ M) into the stirred calorimeter cell (1.43 mL) which contains at the beginning pure solvent. An injection sequence of 127 x 1.5 $\mu$L in 2 min intervals was used. The data were analyzed using MicroCal Origin.

## 2. Schematic illustration of a $K_2$-$K$ aggregation process

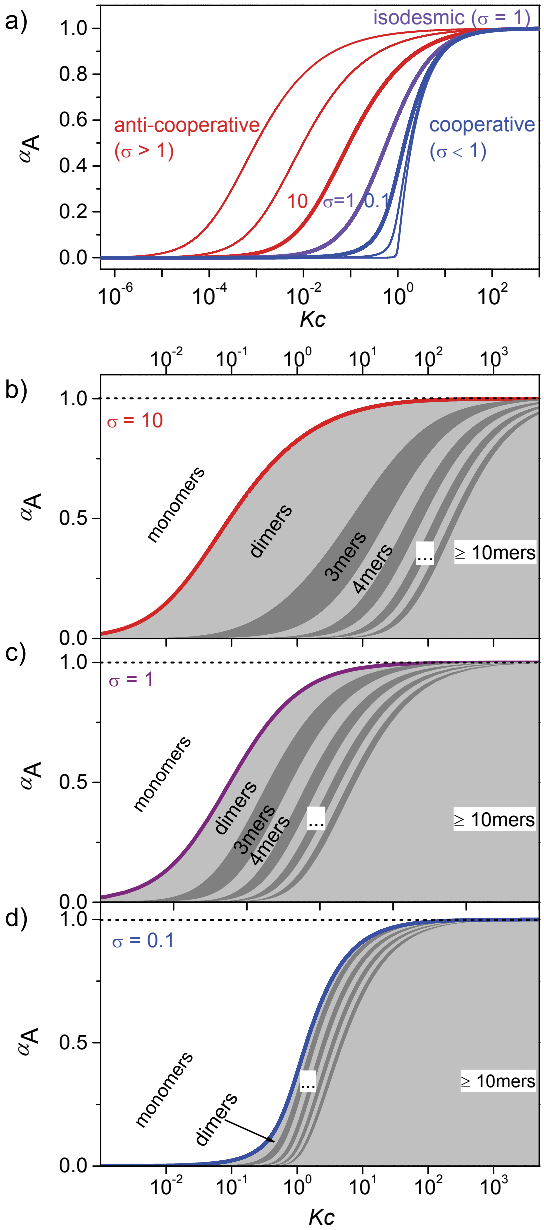


**Fig. S1** a) Schematic illustration of a $K_2$-$K$ aggregation process. Plot of the fraction of aggregated molecules $\alpha_A$ as a function of the normalized concentration $Kc$ with different $\sigma$ values according to eqn (1). For the curves from left to right $\sigma = 10^3$, $10^2$, 10, 1, $10^{-1}$, $10^{-2}$, and $10^{-4}$ were applied.[2] b), c) and d) Oligomer size distribution for representative σ values for anti-cooperative (b, $\sigma = 10$), isodesmic (c, $\sigma = 1$), and cooperative (c, $\sigma = 0.1$) aggregation with a nucleus size of $s = 2$ . In the anti-cooperative case a strong prevalence of the dimer is observed for a large concentration regime, whereas in the cooperative case long oligomers are formed as soon as the critical concentration $Kc$=1 is reached. In the isodesmic case a continuous size distribution can be observed.

## 3. Synthesis of PBI 4

A suspension of the L-alanine functionalized PBI **2**[3] (112 mg, 0.210 mmol, 1 equiv.), 3,5-bis(dodycyloxy)benzylamine **3**[4] (300 mg, 0.630 mmol, 3 equiv.), *O*-(7-azabenzotriazol-1-yl)-*N*,*N*,*N'*,*N'*-tetramethyluronium hexafluorophosphate (HATU) (119 mg, 0.313 mmol, 1.5 equiv.) and catalytic amount of *N*,*N*-diisopropylethylamine (DIPEA) (1.6 mL) was stirred in dichloromethane (20 mL) at room temperature for 16 h. After removal of the solvent in vacuo, the crude product was purified by column chromatography ($SiO_2$, $CH_2Cl_2$/MeOH/$NEt_3$, *v/v/v* = 30:1:0) and recycling GPC. Yield: 137 mg (0.095 mmol, 45%) of a red solid; MW ($C_{92}H_{128}N_4O_{10}$) 1450.02 g/mol; m.p.: 88 – 90 °C; $^1$H NMR (400 MHz, $CDCl_3$): $\delta$ = 8.04 (bm, 10H, perylene protons and N*H*), 6.56 (d, $^4J$ = 2.0 Hz, 4H, Ph-*H*), 6.36 (t, $^4J$ = 2.4 Hz, 2H, Ph-*H*), 5.69 (q, $^3J$ = 6.8 Hz, 2H, C*H*), 4.99 (m, 2H, C*H*$_2$), 4.38 (dd, $^3J$ = 4.0 Hz, $^2J$ = 14.8 Hz, 2H, C*H*$_2$), 4.00 (m, 8H, C*H*$_2$), 1.78 (quint, $^3J$ = 6.8 Hz, 8H, C*H*$_2$), 1.69 (d, $^3J$ = 6.8 Hz, 6H, C*H*$_3$), 1.45 (m, 8H, C*H*$_2$), 1.23 (m, 64H, C*H*$_2$), 0.86 (t, $^3J$ = 6.8 Hz, 12H, C*H*$_3$); HRMS (ESI pos.), calculated for ($[M+H]^+$: 1449.97032, found *m/z* = 1449.97386; calculated for ($[M+Na]^+$): 1471.95227, found *m/z* = 1471.95227; elemental analysis calculated (%) for $C_{91}H_{126}N_4O_{10}$: C 76.11, H 8.84, N 3.90; found: C 75.98, H 8.90, N 3.61; $\lambda_{max}$($CHCl_3$)/nm 528, 491 and 460 ($\varepsilon$ / $M^{-1}$ $cm^{-1}$134000, 81500 and 29900).

## 4. Concentration-dependent $^1$H NMR spectra of PBI 4 in chloroform

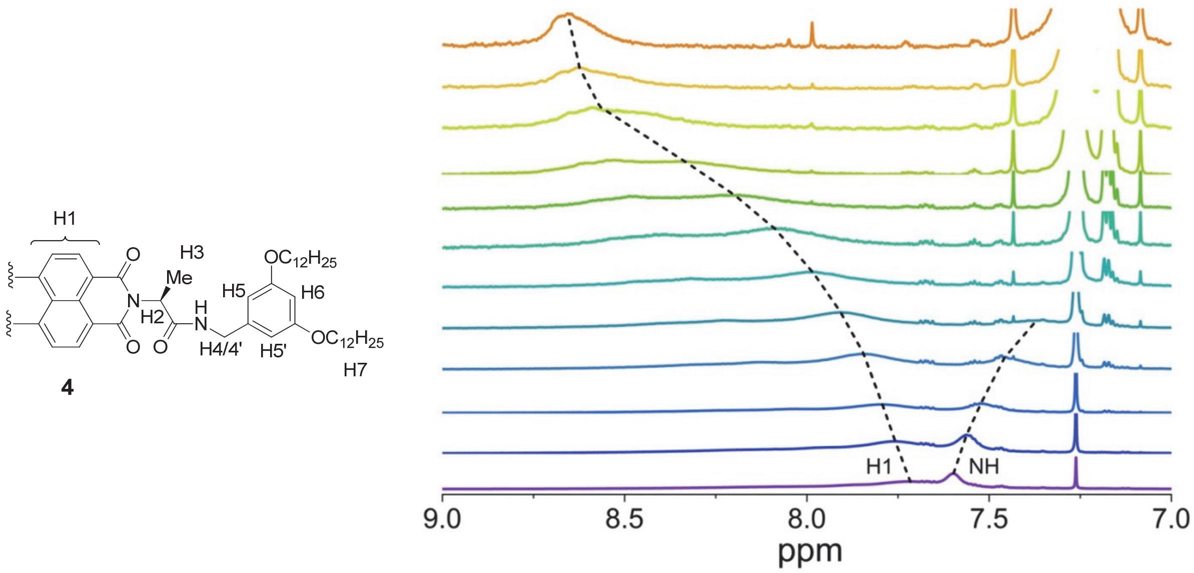


**Fig. S2** Sections (7 to 9 ppm) of concentration-dependent $^1$H NMR spectra of PBI **4** from $6.5 \times 10^{-6}$ M (top) to $1.3 \times 10^{-2}$ M (bottom) in $CDCl_3$ (600 MHz, 298 K).

## 5. Application of the monomer-dimer model[2] on $^{1}$H NMR data

The mathematical description of the equilibrium between monomer (M) and dimer (D) in solution can be written as $M + M \rightleftharpoons D$, and the equilibrium constant $K_2$ can be expressed as:

$$K_2 = [M_2\,]/\,[M]^2 = [M_2\,]/c_M^2 \qquad \text{(S1)}$$

where $[M] = c_M$ is the concentration of the monomers and $[M_2] = c_D$ the concentration of the dimers.

The fraction of monomers $\alpha_M$ (eqn (S2)) and the fraction of aggregated species $\alpha_A$ (eqn (S3)) can be calculated as follows:

$$\alpha_M = c_M\,/c \qquad \text{(S2)}$$

$$\alpha_A = 1 - \alpha_M \qquad \text{(S3)}$$

where $c$ denotes the total concentration.

Considering $c = 2c_D + c_M$ the following equations for $c_M$, $\alpha_M$ and $\alpha_A$ as functions of $K_2$ and $c$ can be derived:

$$c_M = \frac{\sqrt{8K_2c+1}-1}{4K_2} \qquad \text{(S4)}$$

$$\alpha_M = \frac{\sqrt{8K_2c+1}-1}{4K_2c} \qquad \text{(S5)}$$

$$\alpha_A = \frac{4K_2c+1-\sqrt{8K_2c+1}}{4K_2c} \qquad \text{(S6)}$$

Using eqn (S5) and (S6) to describe the change of the chemical shifts with concentration, the dimer model can be fitted to the NMR data of PBI **4** in chloroform in the concentration range $6.5 \times 10^{-6}$ M – $1.3 \times 10^{-}$ M yielding the following results (Fig. S3 and Table S1).

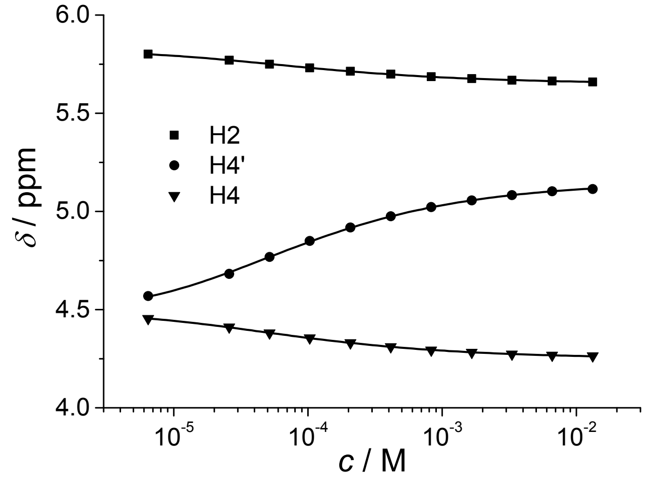


**Fig. S3** Fitting of the concentration-dependent chemical shift ($\delta$) changes of protons H2 and H4/4' to the dimer model by means of nonlinear least-squares analysis as representative examples (correlation coefficient $R^2 = 0.999$).

**Table S1** Dimerization constants ($K_2$) and degrees of aggregation ($\alpha_A$) obtained in the considered concentration range ($6.5 \times 10^{-6}$ – $1.3 \times 10^{-2}$ M) from the best fitting of the chemical shift changes of the protons of PBI **4** in chloroform.

| Protons | H1 | H2 | H3 | H4 | H4' | H5/5' |
|---|---|---|---|---|---|---|
| $K_2$ / $10^4$ $M^{-1}$ | 1.0 ± 0.08 | 1.1 ± 0.04 | 1.0 ± 0.08 | 1.3 ± 0.06 | 1.4 ± 0.05 | 1.2 ± 0.03 |
| $\alpha_A$ [a] | 1 – 98% | 11 – 95% | 11 – 93% | 13 – 94% | 14 – 94% | 12 – 95% |

[a] $\alpha_A$ value at the lowest concentration of $6.5 \times 10^{-6}$ M and the highest concentration of $1.3 \times 10^{-2}$ M.

## 6. FT-IR measurements in chloroform and toluene

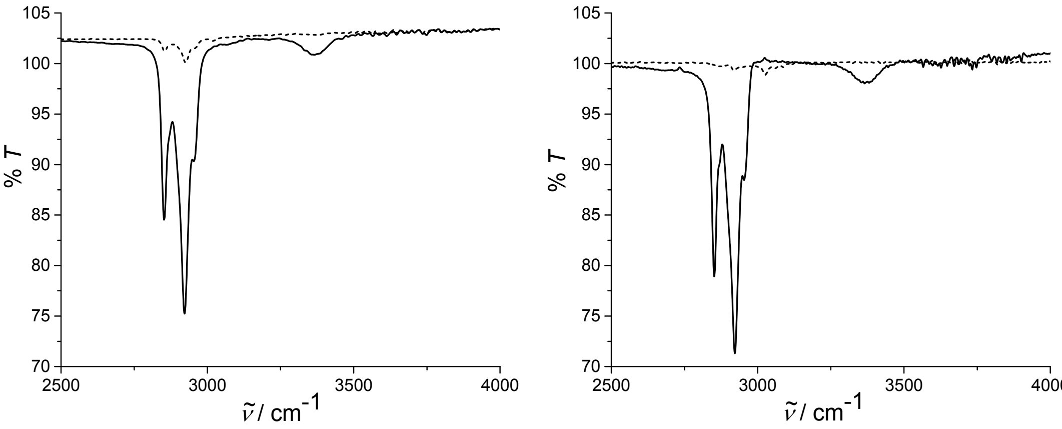


**Fig. S4** FT-IR spectra of PBI **4** at the concentrations of $1.3 \times 10^{-2}$ M (solid line) and $4.1 \times 10^{-4}$ M (dotted line) in chloroform (left) and toluene (right) at 293 K. The spectra of PBI **4** show two sets of signals. The prominent signals at 2853 and 2922 $cm^{-1}$ are assigned to the CH stretching vibrations. The small broad band at 3370 $cm^{-1}$ in chloroform and 3367 $cm^{-1}$ in toluene are attributed to the hydrogen bonded NH stretching vibration. The stretching vibrations of free NH groups which can usually be found in the region between 3500 and 3600 $cm^{-1}$ could not be detected, which means that all NH groups are involved in hydrogen bonds.[5]

## 7. DOSY NMR experiments in chloroform and toluene

The hydrodynamic radius $R$ of PBI **4** is determined from the diffusion coefficient $D$ using the Stokes-Einstein equation (eqn (S7)):

$$D = \frac{k_B T}{6\pi\eta R} \quad \text{(S7)}$$

where $k_B$ is the Boltzmann constant, $T$ the temperature, and $\eta$ the viscosity of the solvent. The diffusion constant in turn results from DOSY NMR measurements.

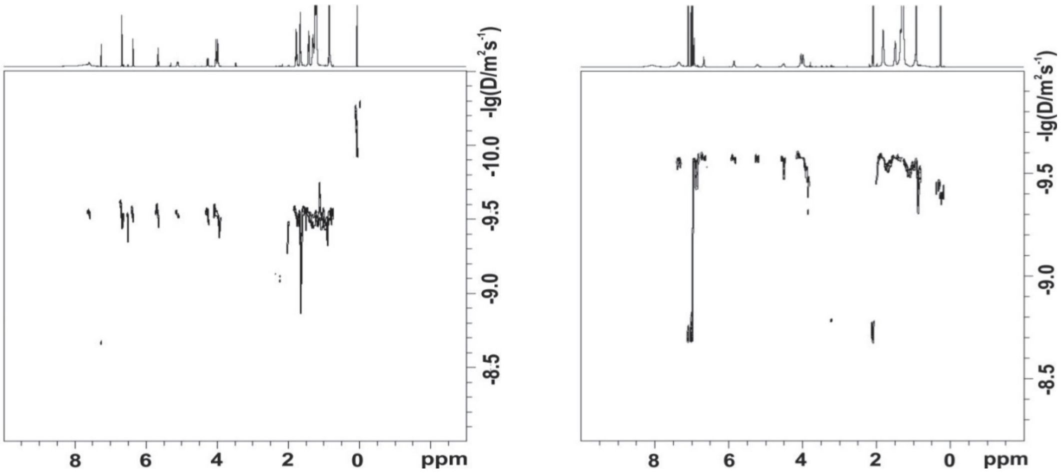


**Fig. S5** DOSY NMR spectrum of PBI **4** at $c$ = 1.3 x $10^{-2}$ M in chloroform (left) and toluene (right) recorded at 600 MHz NMR at 293 K. The diffusion coefficients $D$ [$m^2$ $s^{-1}$] are plotted in a logarithmic scale against the chemical shift $\delta$ [ppm]. The hydrodynamic diameter of 2.6 nm in chloroform and 2.8 nm in toluene were calculated.

### 8. VPO measurements in chloroform

The vapour pressure osmometry (VPO) provides the possibility to measure the total osmolality of physiological fluids and to calculate the molecular mass of compounds in aqueous and organic solutions. The solutions containing solutes have lower vapour pressure than the pure solvent which leads to a vapour pressure difference, and thus to a temperature difference ($\Delta T$) during the measurement. This ΔT is proportional to the number of particles or number of moles dissolved in the solution. By this method concentrations or the molecular mass can be determined. For the determination of the mass a standard has to be used, in our case bezil. In the calibration measurement, we measured voltages at constant temperature of 303 K which are divided by the concentration (molalities) and plotted against the molality of the calibrant. The linear regression leads to the calibration constant $K_{cal}$ as the y-value at $c$ = 0. The values for PBI **4** are plotted correspondingly to the calibration measurement. The $c$ / g $kg^{-1}$ value was obtained by dividing the initial weight of PBI **4** with the weight of the solvent. The extrapolation leads to the $K_{meas}$ value. The molecular weight is determined by the equation:

$$M = K_{cal}/K_{meas} = 2955.6 \text{ g/mol} \quad \text{(S8a)}$$

$N = M/M_{\mathrm{PBI}\mathbf{4}} = 2.04$ (S8b)

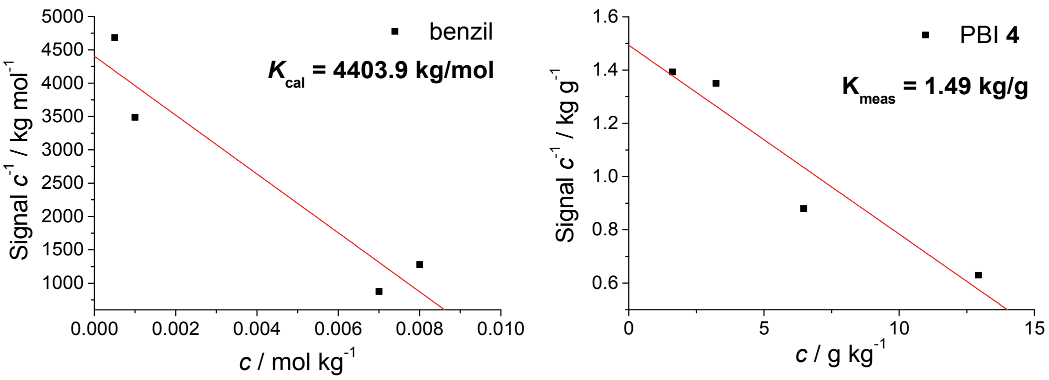


**Fig. S6** VPO measurements of benzil (calibration curve, left) and PBI **4** in chloroform at 303 K ($1.3 \times 10^{-2}$ to $1.7 \times 10^{-3}$ M, right). The *K* values are interpolated y values at x = 0.

## 9. ROESY NMR experiments in chloroform

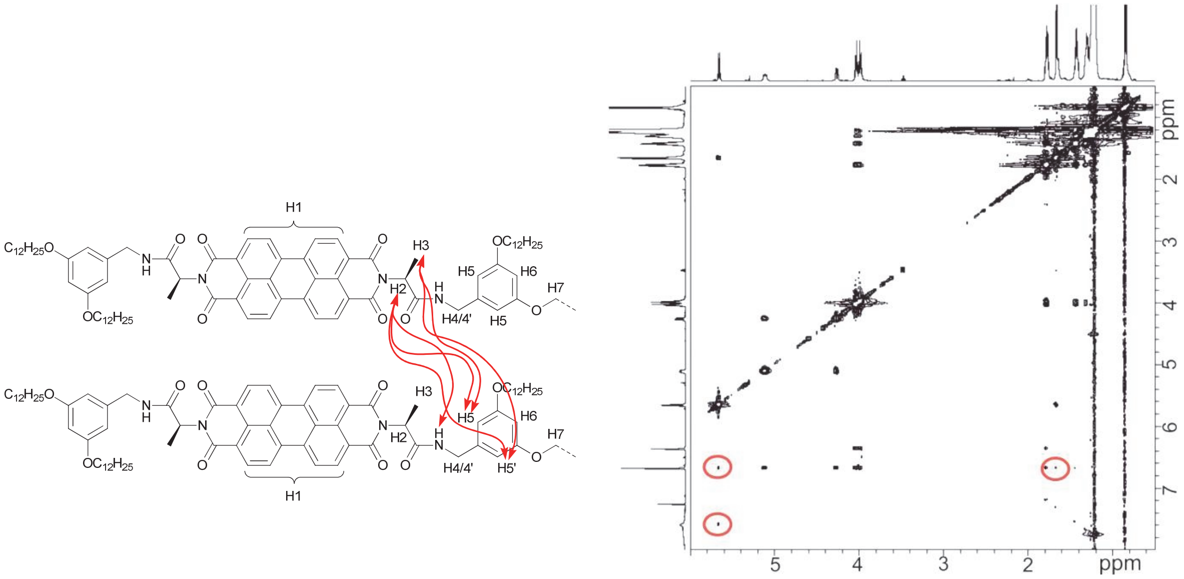


**Fig. S7** Extended part of ROESY NMR spectrum of PBI **4** at $c = 1.3 \times 10^{-2}$ M in chloroform recorded with 600 MHz NMR at 298 K.

## 10. Isothermal titration calorimetry experiments in chloroform

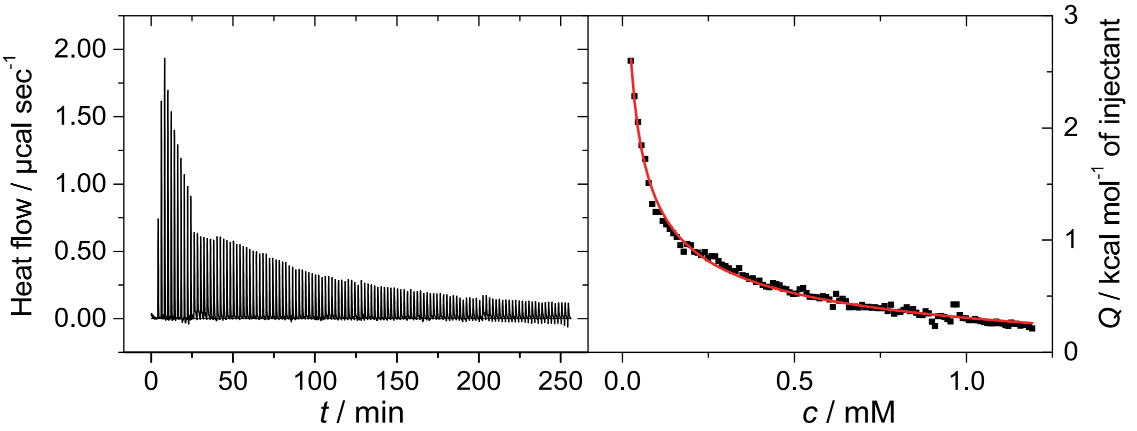


**Fig. S8** Calorimetric data for the dissociation process of the PBI **4** in chloroform. Left: Raw data for injection of PBI **4** with an injection concentration of 7.5 x $10^{-3}$ M into chloroform at 293 K (end concentration of PBI **4** in the cell is 1.1 x $10^{-3}$ M). Right: Integrated injection heats and fit (red line) to the dimer dissociation model with $K_{diss}$ = 0.0968 ± 0.011 mM ($K_{ass} = 1/K_{diss}$ = 1.0 x $10^4$ $M^{-1}$) and (36.5 ± 1.5) kJ $mol^{-1}$.

## 11. Concentration-dependent UV/Vis spectra of PBI 4 in toluene

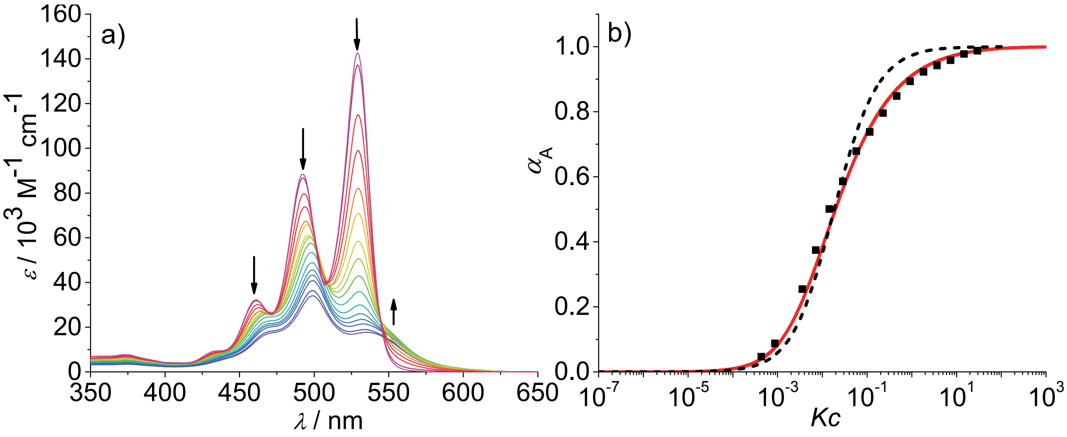


**Fig. S9** a) Concentration-dependent UV/Vis absorption spectra of PBI **4** in toluene ($c$ = 2.0 x $10^{-7}$ – 1.3 x $10^{-2}$ M) at 293 K. Arrows indicate the spectral changes upon increasing the concentration. b) Analysis according to isodesmic (dashed black line) and $K_2$-$K$ (red line) aggregation models, with s = 2, $\sigma$ = 50 and $K$ = 2.20 x $10^3$ $M^{-1}$.

## 12. Conventional $K_2$-$K$ model[2,6]

Taking into account that the equilibrium constant of the dimerization differs from the elongation constant, the isodesmic model has to be modified. With the nucleus of s = 2, the equilibrium is described as follows:

$$M + M \overset{K_2}{\rightleftharpoons} D$$

$$D + M \overset{K_3}{\rightleftharpoons} T$$

$$M_{n-1} + M \overset{K_n}{\rightleftharpoons} M_n$$

$$K_2 \neq K_3 = \ldots K_n = K \tag{S9}$$

By defining $\sigma = K_2/K$, the concentration of n-mer $c_n$ and the total concentration $c$ can be obtained with the following equations:

$$c_n = K_2 K^{n-2} c_M^n = \sigma K^{n-1} c_M^n \tag{S10}$$

$$c = c_M + \sum_{n=2}^{\infty} n\sigma K^{-1} (K c_M)^n = (1 - \sigma) c_M + \frac{\sigma c_M}{(1 - K c_M)^2} \tag{S11}$$

By multiplying eqn (S11) with $K$, eqn (1) shown in the paper is obtained.

## 13. VPO measurements in toluene

$$K_{cal}/K_{meas} = 3068.59 \text{ g/mol} \tag{S12}$$

$$N = 2.12 \tag{S13}$$

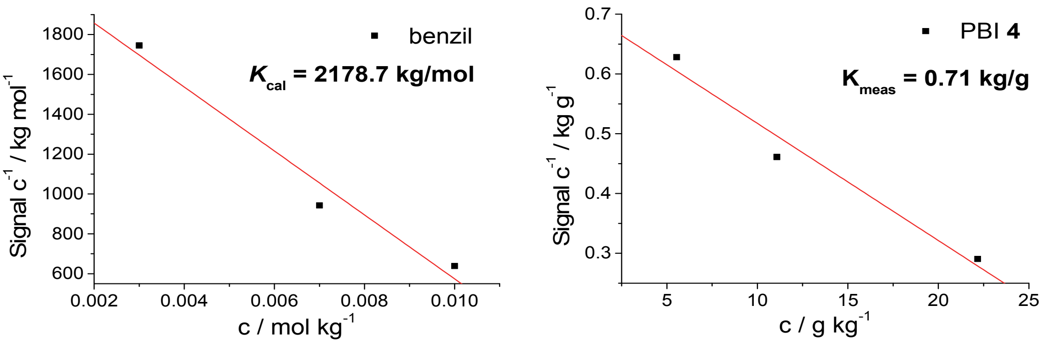


**Fig. S10** VPO measurements of benzil (calibration curve, left) and PBI **4** in toluene at 303 K ($1.3 \times 10^{-2}$ to $1.7 \times 10^{-3}$ M, right). The $K$ values are obtained by interpolating the data to x = 0.

## 14. Analysis of absorption data of PBI 4 in methylcyclohexane at 502 nm

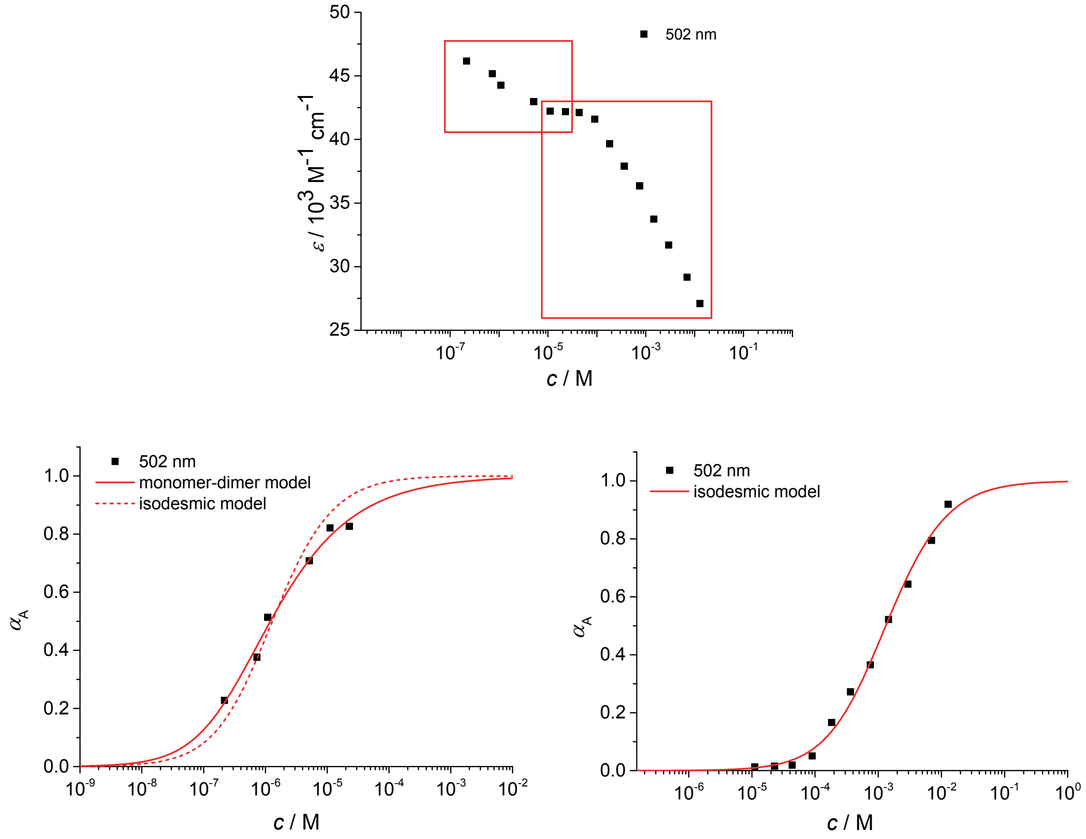


**Fig. S11** a) Concentration-dependent absorption data of PBI **4** in methylcyclohexane at 502 nm. b) Fit of the data to the monomer-dimer model ($c$ = 2.3 x $10^{-5}$ to 2.2 x $10^{-7}$ M, $R^2$ = 0.987); dashed line: isodesmic model for comparison. c) Fit of the data to the isodesmic model ($c$ = 1.3 x $10^{-2}$ to 1.1 x $10^{-5}$ M, $R^2$ = 0.995).

## 15. DOSY NMR experiments in methylcyclohexane

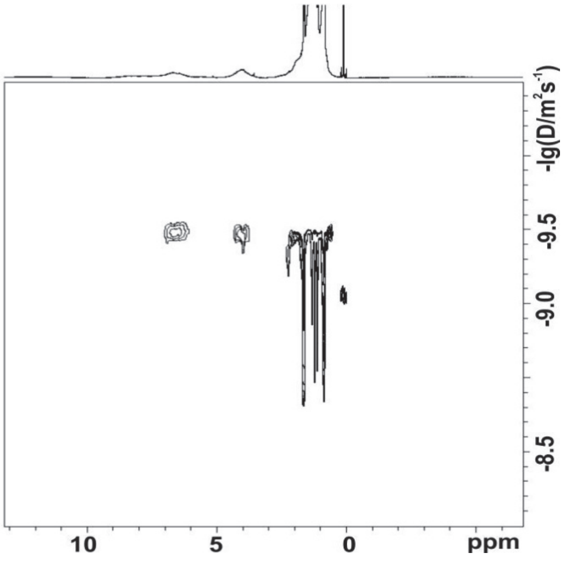


**Fig. S12** DOSY NMR spectrum of PBI **4** at $c = 1.3 \times 10^{-2}$ M in methylcyclohexane recorded with 600 MHz NMR at 296 K. The diffusion coefficients $D$ [$m^2$ $s^{-1}$] are plotted in a logarithmic scale against chemical shift $\delta$ [ppm].

## 16. Absorption spectra of PBI 4 in methylcyclohexane/toluene mixtures

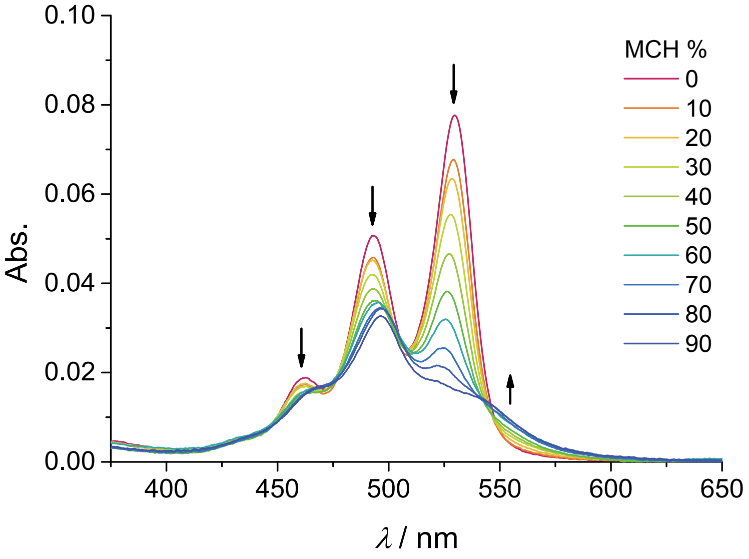


**Fig. S13** Solvent-dependent UV/Vis absorption spectra of PBI **4** (methylcyclohexane content from 0 to 90%) at the concentration of $1.6 \times 10^{-6}$ M recorded at 293 K. Arrows indicate the spectral changes upon increasing methylcyclohexane content.

## 17. Analysis of absorption data of PBI 4 in a methylcyclohexane/toluene (30:70) mixture at 476 nm

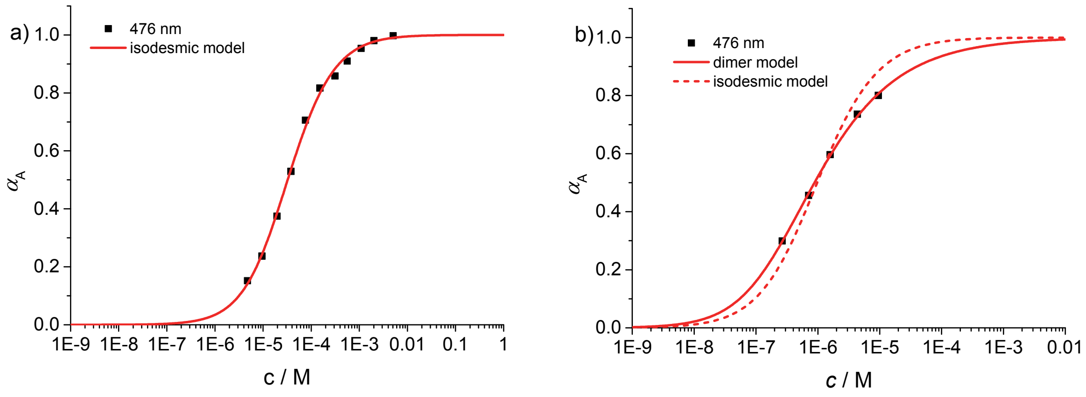


**Fig. S14** a) Fit of the isodesmic model ($R^2$ = 0.998) to the data obtained at the concentration range *c* = 1.0 x $10^{-2}$ to 9.5 x $10^{-6}$ M. b) Fit to the monomer-dimer model ($R^2$ = 0.999) of the data obtained at the concentration range *c* = 9.5 x $10^{-6}$ to 2.7 x $10^{-7}$ M. Dashed line: isodesmic model for comparison.

## 18. $K_2$-$K$-model for anti-cooperative supramolecular polymerization

The $K_2$-$K$ model for anti-cooperative supramolecular polymerization describes an aggregation behaviour where the formation of dimers is favoured by a strong interaction between the involved molecules, e.g. by the formation of H-bonds. As the dimerization leads to a saturation of the bond donating unit, further elongation is disadvantaged due to a much weaker stacking force. Therefore, the elongation constant $K$ is considerably smaller compared to the dimerization constant $K_2$. Such an aggregation behaviour favours the formation of even numbered aggregates. In the following, the equations that describe the aggregation behaviour are derived.

Even numbered aggregates:

Monomers grow into dimers with the dimerization constant $K_2$. The stacking of dimers into even numbered aggregates continues with the elongation constant $K$.

| | | | |
|---|---|---|---|
| $M + M \rightleftharpoons D$ | $[M_2] = K_2 c_M^2$ | (dimer) | (S14) |
| $D + D \rightleftharpoons D_2$ | $[M_4] = K[M_2]^2$ | (tetramer) | (S15) |
| $D_2 + D \rightleftharpoons D_3$ | $[M_6] = K[M_2][M_4] = K^2[M_2]^3$ | (hexamer) | (S16) |

$D_{n-1} + D \rightleftharpoons D_n \qquad [M_{2n}] = K[M_2]^{n-1}[M_2] = K^{n-1}[M_2]^n \qquad$ ((2n)-mer) (S17)

where $[M_{2n}]$ is the concentration of aggregates which contain *2n* monomers. To obtain the number of monomers in an aggregate with a size of *2n* monomers, eqn (S17) has to be multiplied by *2n*.

The concentration of molecules in even numbered aggregates is calculated by performing the sum over all possible *n* starting at *n = 1* (dimer) up to infinity:

$$c_A^{even} = 2 * [M_2] + 2 * 2[M_4] + 2 * 3[M_6] + \cdots + 2n * [M_{2n}] = 2 * \sum_{n=1}^{\infty} n\,[M_{2n}] =$$
$$2 * \sum_{n=1}^{\infty} nK^{n-1}[M_2]^n = \frac{2}{K}\sum_{n=1}^{\infty} nK^n[M_2]^n \qquad \text{(S18)}$$

The sum is evaluated making use of the geometric power series

$$\sum_{n=0}^{\infty} nq^n = \frac{q}{(1-q)^2} \qquad \text{with } q = K[M_2] <. \qquad \text{(S19)}$$

Consequently, the concentration of molecules in an even numbered aggregate is given by:

$$c_A^{even} = \frac{2}{K}\frac{K[M_2]}{(1-K[M_2])^2} = 2\frac{K_2 c_M{}^2}{(1-KK_2c_M^2)^2} \qquad \text{with } K[M_2] < 1 \qquad \text{(S20)}$$

<u>Odd numbered aggregates:</u>

Odd numbered aggregates are constructed either by the elongation of an even numbered aggregate with a monomer or of an odd numbered aggregate with a dimer. In both cases the aggregation process is quantified by the elongation constant *K* describing the weaker interaction.

$D + M \rightleftharpoons M_3 \qquad [M_3] = K[M_1][M_2] = KK_2c_M^3 \qquad$ (trimer) (S21)

$D_2 + M \rightleftharpoons M_5 \qquad [M_5] = K[M_1][M_4] = K[M_2][M_3] = K^2K_2^2c_M^5 \qquad$ (pentamer) (S22)

$D_n + M \rightleftharpoons M_{2n+1} \qquad [M_{2n+1}] = K_2^{n-1}K^{n-1}c_M^{2n-1} = c_M K^n [M_2]^n \qquad$ ((2n+1)-mer) (S23)

The concentration of molecules in odd numbered aggregates (with a size of at least 3 monomers) can be calculated with the help of eqn (S19) by:

$$c_A^{odd} = 3 * [M_3] + 5 * [M_5] + \cdots + (2n+1) * [M_2]^n K^n c_M = \sum_{n=1}^{\infty}(2n+1) * [M_2]^n K^n c_M = 2c_M *$$
$$\sum_{n=0}^{\infty} n * ([M_2]K)^n + c_1 * \sum_{n=0}^{\infty}([M_2]K)^n - c_M = c_M \frac{[M_2]K+1}{(1-[M_2]K)^2} - c_M = \frac{[M_2]Kc_M(3-[M_2]K)}{(1-[M_2]K)^2} =$$
$$\frac{KK_2c_M{}^3(3-KK_2c_M{}^2)}{(1-KK_2c_M{}^2)^2} \qquad \text{with } KK_2c_M{}^2 < 1 \qquad \text{(S24)}$$

The term $-c_M$ has to be added in order to change the lower limit of the summation index *n* from 1 to 0. The total concentration *c* in the system can be calculated in dependence of the monomer concentration $c_M$ by:

$$c = c_M + \underbrace{\frac{2K_2c_M{}^2}{(1-K_2c_M{}^2K)^2}}_{even} + \underbrace{\frac{KK_2c_M{}^3(3-KK_2c_M{}^2)}{(1-KK_2c_M{}^2)^2}}_{odd} \tag{S25}$$

The ratio between even and odd numbered aggregates is given by:

$$\frac{c_A^{odd}}{c_A^{even}} = \frac{Kc_M[M_2](3-[M_2]K)}{(1-[M_2]K)^2}\frac{(1-[M_2]K)^2}{2[M_2]} = \frac{Kc_M(3-[M_2]K)}{2} \tag{S26}$$

For simplification, the abbreviation $K[M_2] = a$ is used. This leads to an expression for the monomer concentration of $c_M = \sqrt{\frac{a}{K_2K}}$ . Insertion of the latter term into eqn (S26) and reformulation leads to:

$$\frac{c_A^{odd}}{c_A^{even}} = \sqrt{\frac{K}{K_D}}\ \frac{\sqrt{a}}{2}(3-a) \tag{S27}$$

The term $\frac{\sqrt{a}}{2}(3-a) < 1$ for all $a < 1$. Therefore, the maximal fraction of molecules in odd numbered aggregates is always smaller than the square root of the aggregation constants:

$$\frac{c_A^{odd}}{c_A^{even}} < \sqrt{\frac{K}{K_2}} \tag{S28}$$

## 19. Fit algorithm to analyse the anti-cooperative supramolecular polymerization

The $K_2$-$K$ model for anti-cooperative supramolecular polymerization is fitted to the data by varying the aggregation constants $K_2$ and $K$. Additionally, the extinctions of the three distinct species, monomer, dimer and oligomer are optimized. The fitting routine is implemented in Labview and optimizes the model parameters globally in order to describe the two dimensional, experimental data set of concentration-and wavelength-dependent extinction $\boldsymbol{data}(\lambda, c)$. The fit algorithm is comparable to our previously reported procedure,[7] the basic steps are:

0. The start values of the aggregation constants $K_2$ and $K$ are chosen manually.
1. An array of size *N* for the monomer concentration $c_M$ is constructed by following way: The

minimum value of the monomer concentration is chosen to be $c_{M,min}$ = 1*10$^{-7}$ M and the maximum monomer concentration for anti-cooperative supramolecular polymerization is given by the aggregation constants $c_{M,max} = \sqrt{\frac{1}{K_2 K}}$ . The maximum monomer concentration is reached asymptotically for high dye concentrations and a small variation of the monomer concentration for a large interval of the total concentration is inherent for anti-cooperative supramolecular polymerization. Therefore, a non-equidistant grid is used for the monomer concentration with large intervals for low dye concentrations, i.e. the first N/2 steps and small steps for high dye concentrations in the second half of the $c_M$ - axis.

- $n = 1 \ldots \frac{N}{2}$: $c_{M,n} = c_{M,min} * \left(\frac{c_{M,max}}{c_{M,min}}\right)^{n-1 / \frac{N}{2}}$ (S29)
- $n = \frac{N}{2} + 1 \ldots N$: $c_{M,n} = c_{M,N/_2} + \frac{c_{M,max} - c_{M,N/_2}}{N/_2} * \left(n - \frac{N}{2}\right)$ (S30)

2. The concentration of molecules in dimers and in aggregates is calculated in dependence of the monomer concentration $c_{M,n}$. The concentration of molecules in dimers is given by

   $$c_D = 2\, K_2 c_M^2 \quad \text{(S31)}$$

   The concentration of molecules in aggregates is the sum of molecules in odd (eqn (S24)) and even (eqn (S22)) numbered aggregates minus the concentration of molecules in dimers:

   $$c_A = c_A^{odd} + c_A^{even} - c_D \quad \text{(S32)}$$

   The total concentration is calculated with eqn (S25).

3. The fraction of molecules in a distinct species is calculated by $\alpha_{M,D,A} = \frac{c_{M,D,A}}{c_T}$ and the total concentration *c* is interpolated to the measured concentrations for comparison.
4. The extinction of each species (monomer, dimer and aggregate) is calculated for the given set of aggregation constants by $\boldsymbol{\varepsilon}_{\boldsymbol{M,D,A}}(\lambda) = \boldsymbol{data}(\lambda, c)\boldsymbol{\alpha}(c)[\boldsymbol{\alpha}^T(c)\boldsymbol{\alpha}(c)]^{-1}$with $\boldsymbol{\alpha}(c) = [\alpha_M\ \alpha_D\ \alpha_A]$.

5. The aggregation constants $K_2$ and $K$ are optimized and step 1 to 5 are repeated until the error between the fitted and measured extinction is minimized.

**20. Analysis of the concentration-dependent UV/Vis spectra of PBI 4 in a methylcyclohexane/toluene mixture of 30:70 by the $K_2$-$K$ model for anti-cooperative supramolecular polymerization**

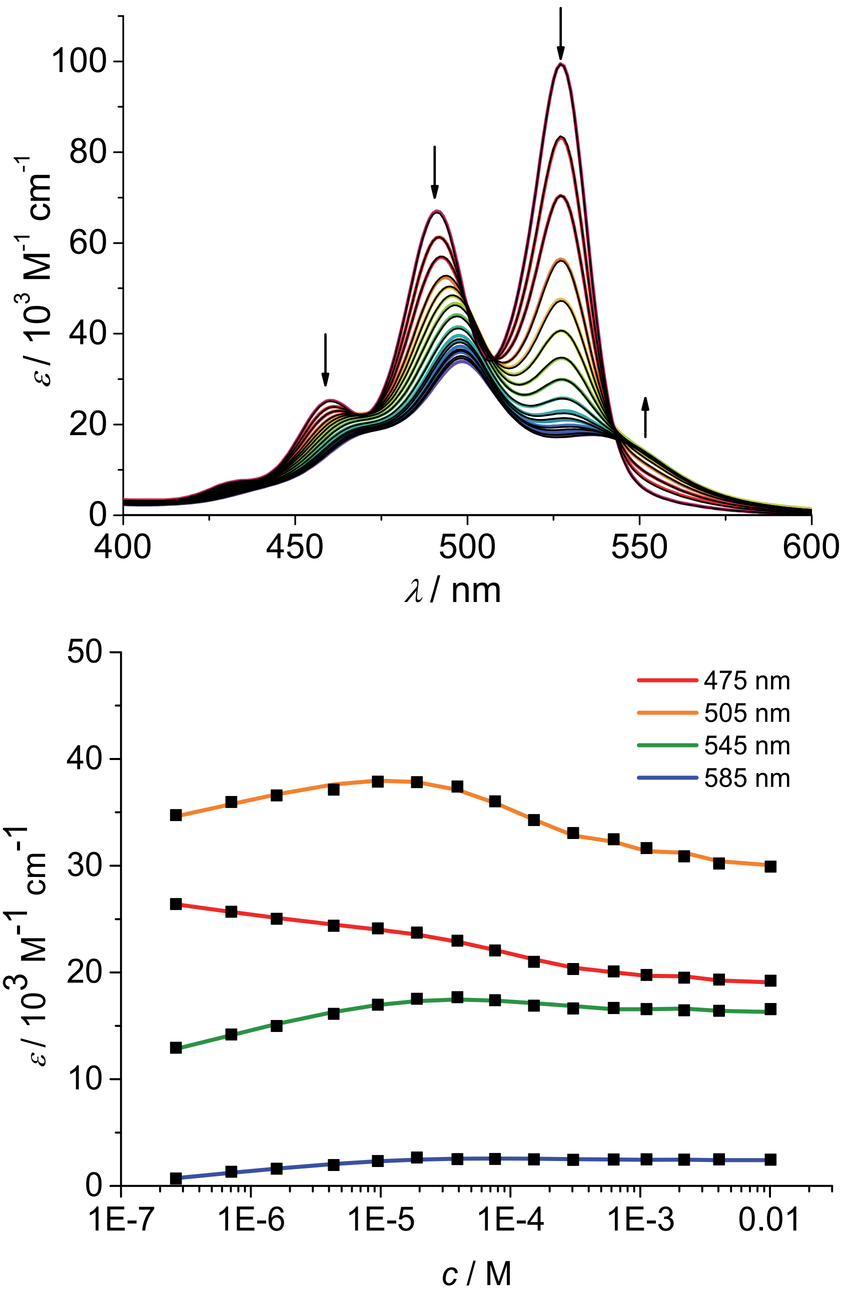


**Fig. S15** Concentration-dependent UV/Vis absorption spectra (coloured lines) and the spectra reconstructed by the new $K_2$-$K$ model for anti-cooperative supramolecular polymerization (black lines) of PBI **4** in a methylcyclohexane/toluene (30:70) mixture (c = 2.7 x 10-7 – 1.0 x 10-2 M) at 293 K (top). Arrows indicate the spectral changes upon increasing concentration. Fit of the $K_2$-$K$ model for anti-cooperative supramolecular polymerization to the concentration-dependent absorption data at various wavelengths (bottom).

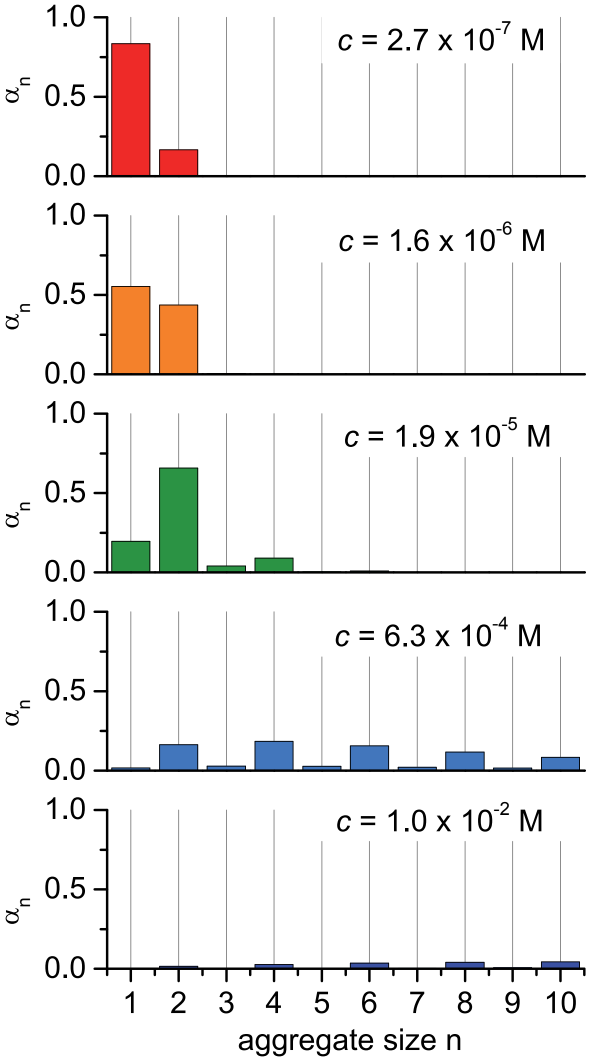


**Fig. S16** Histograms obtained from eqn (S17) and eqn (S23) for five different concentrations in methylcyclohexane/toluene mixture showing the aggregate size distribution up to an aggregate size of ten. A prevalence for even numbered aggregates is observed as soon as the system starts to aggregate.

## 21. $^1H$ NMR and mass spectra of PBI 4

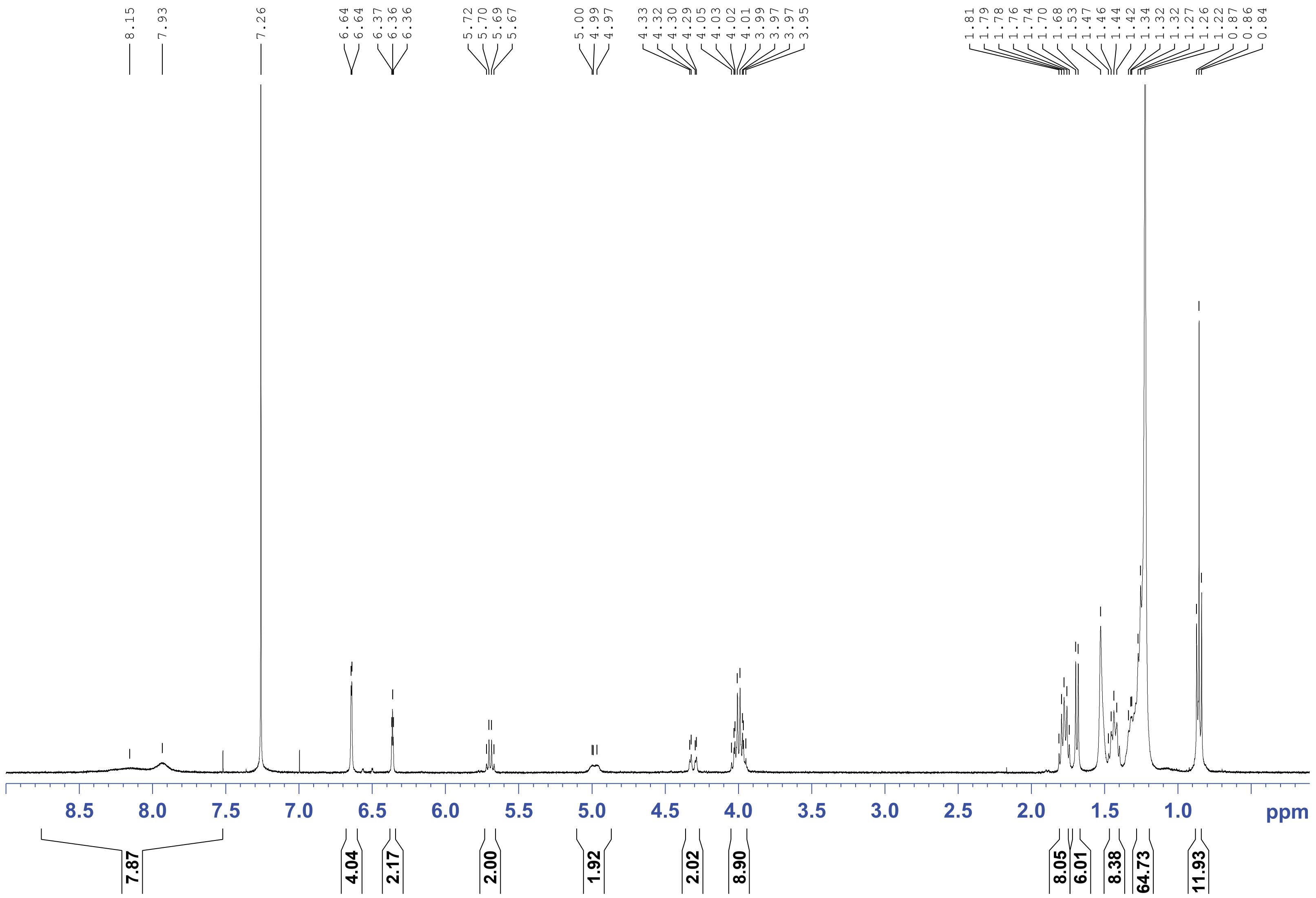


**Fig. S17** $^1H$ NMR spectrum of PBI **4** recorded with 400 MHz NMR in $CDCl_3$ at 298 K.

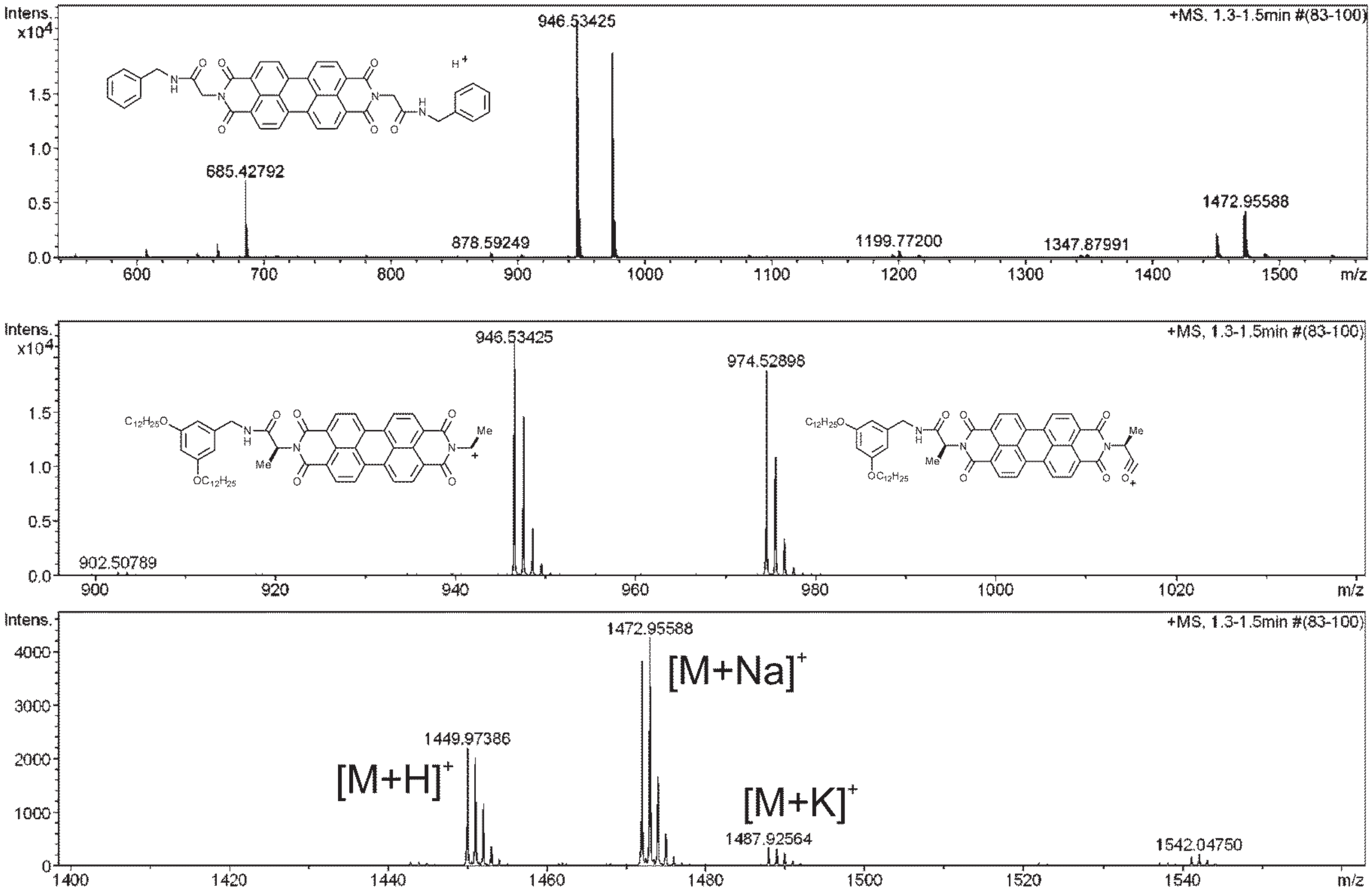


**Fig. S18** HRMS-ESI spectra (positive mode, acetonitrile/chloroform 1:1) of PBI **4**.

## 22. References


1. D. D. Perrin, W. L. F. Armarego and D. R. Perrin, *Purification of laboratory chemicals*, Pergamon Press, Oxford ; New York, 1980.
2. Z. Chen, A. Lohr, C. R. Saha-Möller and F. Würthner, *Chem. Soc. Rev.*, 2009, **38**, 564-584.
3. Y. J. Xu, S. W. Leng, C. M. Xue, R. K. Sun, J. Pan, J. Ford and S. Jin, *Angew. Chem. Int. Ed.*, 2007, **46**, 3896-3899.
4. C. Shao, M. Grüne, M. Stolte and F. Würthner, *Chem. Eur. J.*, 2012, **18**, 13665-13677.
5. A. Syamakumari, A. P. H. J. Schenning and E. W. Meijer, *Chem. Eur. J.*, 2002, **8**, 3353-3361.
6. R. B. Martin, *Chem. Rev.*, 1996, **96**, 3043-3064.
7. F. Fennel, S. Wolter, Z. Xie, P.-A. Plötz, O. Kühn, F. Würthner, S. Lochbrunner, *J. Am. Chem. Soc.,* 2013, **135**, 18722-18725.
